# Chaperone-Dependent Mechanisms as a Pharmacological Target for Neuroprotection

**DOI:** 10.3390/ijms24010823

**Published:** 2023-01-03

**Authors:** Mikhail V. Voronin, Elena V. Abramova, Ekaterina R. Verbovaya, Yulia V. Vakhitova, Sergei B. Seredenin

**Affiliations:** Department of Pharmacogenetics, FSBI “Zakusov Institute of Pharmacology”, Baltiyskaya Street 8, 125315 Moscow, Russia

**Keywords:** chaperones, Sigma1R, BiP, protein folding, ER stress, unfolded protein response, IRE1α, neurodegenerative diseases, neuroprotection

## Abstract

Modern pharmacotherapy of neurodegenerative diseases is predominantly symptomatic and does not allow vicious circles causing disease development to break. Protein misfolding is considered the most important pathogenetic factor of neurodegenerative diseases. Physiological mechanisms related to the function of chaperones, which contribute to the restoration of native conformation of functionally important proteins, evolved evolutionarily. These mechanisms can be considered promising for pharmacological regulation. Therefore, the aim of this review was to analyze the mechanisms of endoplasmic reticulum stress (ER stress) and unfolded protein response (UPR) in the pathogenesis of neurodegenerative diseases. Data on BiP and Sigma1R chaperones in clinical and experimental studies of Alzheimer’s disease, Parkinson’s disease, amyotrophic lateral sclerosis, and Huntington’s disease are presented. The possibility of neuroprotective effect dependent on Sigma1R ligand activation in these diseases is also demonstrated. The interaction between Sigma1R and BiP-associated signaling in the neuroprotection is discussed. The performed analysis suggests the feasibility of pharmacological regulation of chaperone function, possibility of ligand activation of Sigma1R in order to achieve a neuroprotective effect, and the need for further studies of the conjugation of cellular mechanisms controlled by Sigma1R and BiP chaperones.

## 1. Introduction

Alzheimer’s disease (AD), Parkinson’s disease (PD), amyotrophic lateral sclerosis (ALS), and Huntington’s disease (HD) are the main neurodegenerative diseases with a chronic progressive course that jeopardize public health and carry severe economic burden [1].

AD, the most common neurodegenerative disease, showed a 115% increase in disability-adjusted life years (DALYs), a 146% death rise, and a 119% increase in prevalence from 1990 to 2017. For PD, the second most common neurodegenerative disease, correspondent values are 113%, 130%, and 128% [2,3]. Epidemiological data from the United States show that nearly 5 million of Americans suffer from AD [4]. According to [5] since 2000 the number of deaths from AD in the USA increased 145.2% by 2019. Disappointing statistics show that by 2025 in the USA, the number of people aged 65 and older with AD is projected to reach 7.2 million. Over 7 million people worldwide suffer from PD. The incident number of PD in 2019 increased 159.7% since 1990 [6]. Today, 1 million of Americans live with PD [4]. In the USA the prevalence of PD among patients older than 45 years will rise to approximately 1.2 million by 2030 [7]. The number of ALS cases is projected to increase globally from 222,801 in 2015 to 376,674 in 2040 [8]. Despite the relatively low incidence approximately 50% of patients diagnosed with ALS have a life expectancy of 30 months after the onset of symptoms [9]. Currently, 30,000 of Americans suffer from ALS [4]. HD cases show similar prevalence [10].

The research and treatment development for neurodegenerative disorders cost a huge amount of money [11]. In the USA, AD alone is responsible for an economic burden of USD 352 billion. Losses from PD in 2017 were estimated at USD 54.7 billion and are projected to rise to USD 83.4 in 2037. According to [12], in 2020, the total annual care-cost of AD and related dementias, PD and motor neuron diseases, specifically ALS and spinal muscular atrophy (SMA), reached USD 655 billion.

The current understanding of neurodegenerative diseases’ pathogenesis is summarized in numerous reviews [13,14,15,16,17,18,19,20,21]. There are several damage mechanisms mainly responsible for neurodegeneration at the cellular level [13]. They include disruption of protein folding and proteotoxicity [14], prolonged ER stress [15,16], mitochondrial dysfunction and ROS overproduction [17,18], Ca^2+^ disbalance [19], excitotoxicity [20], microglia activation and neuroinflammation [21]. Modern studies pay close attention to genome instability contribution to the pathogenesis of neurodegenerative diseases and its relationship with impaired protein quality control [22,23]. The mentioned mechanisms of cell damage are closely linked and form vicious circles of neurodegeneration [13,18,22,24].

The existing approaches to pharmacotherapy of neurodegenerative diseases are mainly based on the known contribution of neurotransmitter system disorders to their pathogenesis and are covered in a number of reviews [25,26,27]. Acetylcholinesterase inhibitors donepezil and rivastigimine are used to treat AD. Approaches to AD therapy based on the reduction in Amyloid-β (Aβ) peptide levels have not shown sufficient efficacy yet [28]. Perspective directions of AD pharmacotherapy development are comprehensively considered in the review [29]. Levadopa, dopamine receptor agonists (pergolide, bromocriptine), amantadine, which exhibit dopamine-mimetic and NMDA receptor antagonist properties, MAO-B and COMT inhibitors, along with anticholinergic drugs are most widely used for PD pharmacotherapy. Until recently, only two drugs with unspecified mechanisms of action were approved for use in ALS patients—riluzole and edaravone. In 2022 another drug with an unknown mechanism of action based on a combination of sodium phenylbutyrate and taurursodiol (relyvrio, PB-TURSO), was approved for ALS treatment by FDA. Reserpine and dopamine receptor antagonists remain the drugs of choice for HD treatment [25,26,27].

Currently, main neurodegenerative diseases are incurable, and pharmacotherapy is aimed at alleviating neurological symptoms [30]. Therefore, the search for new pathogenetic approaches to treatment is crucial. In these terms, gene and stem cells therapy methods seem promising [31,32]. However, considering that the etiopathogenetic basis of neurodegenerative diseases emerge from a disruption of proteins’ native conformation maintenance processes [33,34,35], approaches based on pharmacological regulation of chaperone function are relevant.

From the pharmacological point of view, the description of the chaperone Sigma1R as a protein capable of ligand activation, is very promising [36,37,38,39,40]. In this review, we have attempted to characterize the role of chaperones in the pathogenesis of neurodegenerative diseases and chaperone-dependent mechanisms of neuroprotection in order to identify approaches to pharmacotherapy of neurodegenerative diseases based on the chaperone function regulation.

## 2. Chaperone-Dependent Mechanisms of Proteostasis Maintenance

### 2.1. Mechanisms of ER Stress and Unfolded Protein Response

Functional activity of healthy cells in dynamic changes of endogenous and exogenous factors is possible under the adaptive control of concentration, conformation and localization of certain proteins specific for cellular phenotype, in other words, at maintained proteostasis [41,42,43]. Quantitatively, proteostasis is derived from the balance of protein synthesis, transport, and degradation, while chaperones play a key role in providing proteins with a stable native conformation. Chaperones, in addition to de novo folding, perform protein refolding, prevent protein aggregation and disaggregation [44,45].

Excessive protein supply to the ER and/or disruption of folding causes accumulation of unfolded or misfolded proteins in the ER lumen, which is referred to as ER stress [34]. The emerging events are pertinently illustrated by cases of familial neurodegenerative disorders in which ER stress is promoted by mutations that cause changes in the amino acid sequence of proteins [46,47]. It is shown that ER stress contributes to the disruption of ATPase activity of chaperones (foldases) and oxidative protein modification [44,48], caused by an elevated ROS production both in mitochondria [49,50] and in the ER due to the activation of oxidative protein folding [51,52,53,54]. A decrease in Ca^2+^ levels in the ER lumen plays an important role in the triggering of ER stress [55,56,57].

Cells respond to ER stress by activation of signaling cascades aimed at normalization of protein folding and restoration of proteostasis, known as the unfolded protein response (UPR). Current studies of the UPR activation and signal transduction have been comprehensively systematized in a number of reviews [34,58,59,60,61]. Briefly, UPR includes two main phases—adaptive and pro-apoptotic, which is triggered when proteostasis cannot be restored. The main mechanisms of the adaptive UPR phase are an overall translation decrease along with the activation of a number of expression factors and upregulation of chaperone genes and antioxidant defense proteins [33,34,58,62]. Adaptive UPR also includes processes of endoplasmic reticulum-associated protein degradation (ERAD) and autophagy [44,59,63]. BiP (GRP78, HSPA5), the major chaperone of ER lumen, plays a key role in the UPR regulation [64,65].

### 2.2. BiP Chaperone Contribution to UPR Regulation

The UPR process is triggered after following ER stress sensors have been activated: serine/threonine-protein kinase/endoribonuclease IRE1 (IRE1α), 2-alpha kinase 3 (PERK) eukaryotic translation initiation factor, and ATF-6 alpha (ATF6) acylin AMP-dependent transcription factor. All these ER stress sensors are believed to be inactive in their normal state, forming a complex with BiP [64,65]. Accumulation of unfolded proteins or decreased Ca^2+^ level in the ER promotes dissociation of BiP from ER sensors and activation of the latter [34,59,60,61,66]. It is not fully understood whether this complex dissociation happens due to the competition of unfolded proteins with luminal domain sequences of sensors for substrate-binding domain (SBD) of BiP chaperone or is a result of allosteric regulation of the nucleotide binding domain (NBD, ATPase domain) of BiP after chaperone binding to an unfolded protein. There are data indicating the possibility of direct interaction of luminal domains of ER stress sensors with unfolded proteins [66]. For a review, see [34,59].

PERK and the most evolutionarily conserved ER stress sensor IRE1α [67] are type I transmembrane protein kinases that are activated in a similar manner. After dissociation from BiP, luminal domains of IRE1α and PERK are capable of homodimerization and homooligomerization followed by transautophosphorylation of C-terminal cytoplasmic domains [34,66,68,69].

Phosphorylated dimers of IRE1α cytoplasmic domains acquire the necessary conformation for RNAase activity and excise a 26-nucleotide intron from the mRNA encoding the X-box-binding protein 1 (XBP-1) transcription factor. Such splicing ensures the synthesis of the active transcription factor XBP-1s, capable of enhancing the expression of chaperone genes (including BiP), proteins regulating proteostasis, lipidogenesis, and inflammation [70,71,72,73,74]. More details on the role of XBP-1s in the regulation of target gene expression can be found in the review [75]. On the other hand, IRE1α cytosolic domain RNAse activity provides regulated IRE1-dependent decay (RIDD) of mRNAs and miRNAs, thereby reducing protein load and modulating epigenetic regulation [76,77]. Activation of IRE1α-dependent TRAF2-ASK1-JNRs signaling pathway enhances autophagy [63] without UPR conversion to pro-apoptotic phase [78].

Transautophosphorylation of PERK C-terminal cytoplasmic kinase domain causes phosphorylation of eukaryotic translation initiation factor 2 subunit-α (eIF2α). This reversible chemical modification prevents the formation of the 80S ribosomal complex and protein synthesis [79,80]. At the same time, phosphorylation of eIF2α causes an enhanced expression of ER stress response proteins due to the presence of upstream open reading frames (uORF) in 5′ untranslated regions and internal ribosomal entry sites (IRESs) that promote cap-independent translation of certain mRNAs [81]. IRES sequences have been detected in the mRNA of BiP [82], and transcription factors such as ATF4 [83]. In turn, PERK-dependent activation of ATF4 enhances the expression of BiP chaperone [84] and other genes involved in the protein synthesis and folding, redox-homeostasis, and autophagy [34].

ATF6 is a type II transmembrane protein. Activation of the full-length transcription factor ATF6 (ATF6p90) in response to ER stress occurs after its dissociation from BiP, migration to the Golgi apparatus and cleavage by proteases to form ATF6p50, which translocates into the nucleus and activates the expression of target chaperone genes, ERAD proteins and other UPR effectors [85,86]. Similarly to ATF4, the ATF6 activation has been shown to enhance BiP expression [87,88,89]. In turn, BiP expression enhancement promotes retention of ATF6 in the ER and attenuation of the ATF6 activation [90].

Tunicamycin (an inhibitor of *N*-glycosylation) or thapsigargin (an inhibitor of intracellular SERCA-type Ca^2+^ pumps) are most commonly used as ER stress inducers in vitro [91,92,93,94,95,96,97,98,99,100,101,102,103,104,105]. Elevated BiP levels in cells during the ER stress modeling are considered an UPR marker [91,92,93,94,95,96,97,98,99,100,101,102,103,104,105,106], which is consistent with phosphorylation of IRE1α, PERK, eIF2α, increase in the expression of XBP-1 and in the level of activated ATF6p50 [91,107,108]. Thus, BiP acts as a regulator and effector of the ER-dependent signaling, forming the UPR feedback mechanism.

### 2.3. Sigma1R Chaperone Contribution to UPR Regulation

The Sigma1R chaperone, which has a binding site for endogenous and exogenous ligands [36,37,38], plays an important role in the UPR regulation [109]. Sigma1R is a 223 amino acid-long transmembrane protein. Sigma1R functions are largely determined by its preferential expression in mitochondria-associated membranes (MAMs) of the ER, which form mitochondria-ER contacts (MERCs) and along with the outer mitochondrial membrane provide a cellular interface that ensures energy and IP_3_R3-dependent Ca^2+^ homeostasis of the cell [107,108,110,111]. MAMs are dynamic lipid rafts rich in cholesterol and sphingolipids that cover approximately 5 to 20% of the mitochondrial surface [112,113,114]. The thickness and area of MERCs vary depending on the cell type and metabolism stage [115,116]. More than 100 MERCs-resident proteins have now been shown to ensure many biochemical processes, including Ca^2+^ transport from the ER to mitochondria, autophagosome formation, and phospholipid metabolism [114,116,117]. MERCs perform an important function in maintaining local redox homeostasis, in which regard MAMs are exposed to ROS formed in both mitochondria and the ER [52,118]. The UPR process depends on the morphology and functional activity of MAMs, where, besides Sigma1R and BiP chaperones, ER stress sensors IRE1α and PERK are localized [108,119,120].

The key role of Sigma1R in the regulation of spatial organization and functions of MERCs and MAMs has been established [121,122,123]. Silencing of *Sigmar1* or an antagonistic effect on the chaperone causes at least a 2-fold decrease in the number of MAMs compared to the control [124]. This process is associated with lipid raft alterations, aggregation of Sigma1R, weakening of its chaperone function towards IP_3_R3, degradation and destruction of IP_3_R3-GRP75-VDAC1 functional tethering complex, disruption of Ca^2+^ entry into mitochondria and ATP synthesis [117,124,125,126,127,128].

Sigma1R forms a Ca^2+^-sensitive complex with BiP chaperone, probably through interaction with the latter’s NBD domain [107,129]. This complex dissociates in vitro within 60 min under ER stress simulation conditions or within 30 min if exposed to Sigma1R agonists [107]. The dissociation promotes activation of both chaperones [107,129,130,131,132] and folding of target proteins [39,64]. Sigma1R p.E102Q (c.304G > C p.Glu102Gln rs387906829) mutation eliminates the ability of compounds with agonist properties to cause dissociation of the Sigma1R-BiP complex [107,132], indicating that Sigma1R chaperone activity depends on the protein-ligand interaction. In MAM, Sigma1R also interacts with IRE1α, regulating its stability and phosphorylation level under ER stress [108,133]. Sigma1R expression and stability depend on the nature of ER stress inducers [107]. Additionally, *Sigmar1* gene activity regulates the UPR cell signaling initiation [107,108,133,134,135]. Activated by ER stress or ligands, Sigma1R is capable of translocation as a part of lipid domains from the MAM to the cytoplasmic and nuclear cell membranes [107,136]. Sigma1R has been shown to interact with and regulate the functional activity of ion channels, receptors, and enzymes involved in the pathogenesis of neurodegenerative diseases [38,39,137,138,139].

Thus, Sigma1R physiological functions have a direct effect on endogenous mechanisms of neuroprotection. For example, activated Sigma1R reduce cytotoxicity of glutamate, quinolinic acid, and H_2_O_2_ [140], can regulate intracellular Ca^2+^ balance, restore mitochondrial function, and decrease ROS production [141,142,143]. A number of authors conclude that Sigma1R plays a physiological role in the regulation of brain plasticity [144,145]. Sigma1R role in suppressing glia activation and neuroinflammation was shown [146]. Autophagy is a fundamental process of maintaining cellular homeostasis aimed at degradation and clearance of misfolded proteins and damaged cell organelles. Autophagy downregulation is a typical feature of the pathogenesis of a number of neurodegenerative diseases [147,148]. Sigma1R deficiency contributes to the accumulation of protein aggregates and impairs autophagosome clearance, whereas chaperone activation increases autophagic activity, which is associated with neuroprotective effects [148,149,150,151].

Conversely, cellular damage leading to a decrease in the functional activity of Sigma1R and BiP weakens cytoprotection [94,107]. In the following sections, the contribution of Sigma1R and BiP chaperone activity to the pathogenesis of major neurodegenerative diseases is discussed with reference to clinical and experimental data.

## 3. Alzheimer’s Disease

Clinically, AD manifests as progressive dementia. Apathy and depression are often the primary symptoms. The clinical pattern of the disease is defined by the lesion of the hippocampus and areas of the cerebral cortex responsible for memory, language and thinking, which is typical for AD. At the cellular level, the accumulation of the Aβ plaques outside neurons and twisted strands of the protein Tau (tangles) inside neurons are markers of the disease. AD is subdivided into familial AD (FAD) and sporadic AD (SAD) and depending on the onset before or after 65 years of age into early onset (~10% of patients) and late-onset AD (~90% of patients). Early onset AD has a genetic etiology in almost 100% of cases. The genetic etiology of late-onset AD is detected in over 70% of cases. Autosomal dominant FAD is mostly caused by mutations in the presenilin 1 (*PSEN1*, phenotype MIM 607822), presenilin 2 (*PSEN2*, phenotype MIM 606889), and amyloid beta precursor protein (*APP*, phenotype MIM 104300) genes [5,152,153,154,155,156].

### 3.1. Sigma1R Chaperone in the Pathogenesis of Alzheimer’s Disease

#### 3.1.1. Relation of Sigma1R to AD Pathogenesis in Clinical Trials

Studies on the contribution of SNPs in the *SIGMAR1* to the occurrence of AD are mainly devoted to rs1800866, the C allele of which is associated with an increase in γ-secretase cleavage of APP [157]. However, an ambiguous role of rs1800866 in the predisposition to AD has been shown in various human populations [158,159,160].

Western blot revealed a decrease in Sigma1R levels in postmortem cortical tissue of AD patients [161]. These results are consistent with positron emission tomography (PET) data, showing reduced distribution of cortical Sigma1R in the early phase of AD [162]. In pyramidal cells in the CA1 area of the hippocampi of AD patients, a decrease in ligand binding of Sigma1R was also found [163]. However, a study by A. Yamoah et al. found elevated levels of Sigma1R in hippocampal tissue homogenates and subicular neurons of postmortem brains of patients with mild to moderate AD [164]. In human AD neurons containing small granular misfolded hyperphosphorylated Tau (p-Tau) elevated Sigma1R immunoreactivity and chaperone colocalization with p-Tau aggregates were also detected. Granulovacuolar degeneration of neurons is a feature of AD pathology, which positively correlates with Tau phosphorylation and precedes the formation of neurofibrillary tangles [165]. It was found that such neurons are characterized by a decreased content of Sigma1R [164].

**Figure 1:**
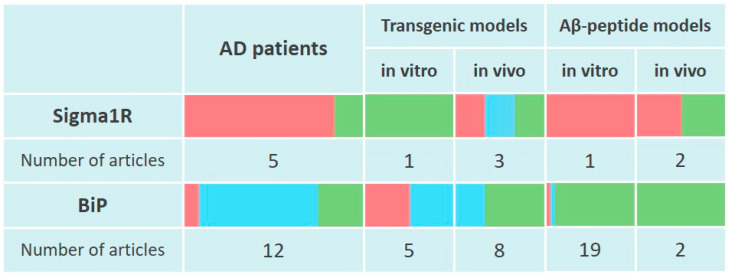
Changes of Sigma1R and BiP chaperones expression in the pathogenesis of Alzheimer’s disease. The table reflects experimentally recorded changes in the expression of Sigma1R and BiP chaperones, denoted in relative units, according to Section 3.1 and Section 3.2. To visualize mRNA or protein levels following markers were used: green—content increased, blue—content did not change, red—content decreased.

Thus, cortical regions of AD human brains show decreased levels of Sigma1R chaperone along with its ligand binding ability. At the same time, current data indicate an adaptive enhancement of Sigma1R expression in the hippocampus during the early stages of AD (Figure 1).

#### 3.1.2. Sigma1R and the Effects of the Chaperone Regulation in AD Modelling by Aβ Peptides Administration

Exposure of Neuro2a cells to Aβ_25–35_ for 48 h attenuates basal expression of Sigma1R and increases BiP level. According to the study results, Aβ_25–35_ promotes ubiquitination and degradation of Sigma1R [166]. Similarly, intracerebroventricular (ICV) injection of Aβ_1–42_ into ICR mice after 22 days leads to a decrease in Sigma1R expression in the hippocampus along with a twofold increase in the Morris water maze test escape latency [167]. However, ICV administration of Aβ_1–42_ into C57BL/6 mice increases the level of Sigma1R in the brain in 14 days. In this experimental model, the Sigma1R agonist PRE-084 stimulated hippocampal cell proliferation and differentiation, which was combined with an even greater increase in Sigma1R content [168].

*Sigmar1* knockout mice are characterized by numerous disorders typical for neurodegenerative diseases [40]. A number of studies have analyzed the effects of chaperone Sigma1R activity regulation on Aβ production and disorders caused by exogenous Aβ peptides. *Sigmar1* knockdown using siRNA in mouse primary hippocampal cultures has itself been shown to cause an increase in caspase-3 activity as well as degeneration of neurons and astrocytes 16 h after treatment [161]. The regulation of *Sigmar1* or Sigma1R protein activity under conditions of AD modeling by Aβ peptides leads to ambiguous results. Pre-injection of the Sigma1R antagonist NE-100 for 7 days or knockout of *Sigmar1* reduced the effective doses of Aβ_25–35_ inducing alteration of spatial working memory and long-term memory in C57Bl/6 mice 6 and 9 days after ICV injection. Sigma1R inactivation also contributed to the Aβ_25–35_-induced increase in lipid peroxidation and the expression of proapoptotic protein Bax, at the same time decreasing BDNF content in mice hippocampus [169]. It is known that proapoptotic Bax expression is also activated under ER stress conditions. Moreover, Bax has the ability to bind to the cytosolic domain of activated IRE1α, presumably stabilizing IRE1α in the active state [91]. It is possible that the ability of Sigma1R to prevent apoptosis during ER stress modeling [108] is related to the chaperone activity of Sigma1R towards IRE1.

On the other hand, knockdown of endogenous *SIGMAR1* in SK-N-MC neuronal cells resulted in a more than 4-fold decrease in γ-secretase activity, which supports the further search for γ-secretase inhibitors that effectively reduce toxic Aβ production [170]. In the in vivo AD model based on Aβ_25–35_ toxicity, protective effects of reducing *Sigmar1* gene activity or antagonizing the chaperone have also been shown. In *Sigmar1^+/−^* mice 12 days after ICV injection of Aβ_25–35_ there was no significant disturbance of short-term spatial memory, apoptosis level decreased, while the survival rate of hippocampal pyramidal neurons increased compared to wild-type (WT) animals. The Sigma1R antagonist NE-100 caused similar outcomes. The authors of the study attribute this result to the reduction in Sigma1R-dependent phosphorylation of the NMDA receptor NR2B subunits and prevention of excessive Ca^2+^ current into neurons [171]. The in vivo effects of NE-100 shown in the cited study are consistent with its ability to prevent tunicamycin-induced cell death and reduce proapoptotic DNA damage-inducible transcript 3 protein (C/EBP-homologous protein, CHOP) levels in murine hippocampal HT-22 cells. It was shown that these effects of NE-100 are associated with an increase in BiP expression probably due to ATF6 activation [172].

Along with the above-mentioned publications showing the protective effect of NE-100, numerous studies indicate the neuroprotective properties of Sigma1R ligands with agonist activity in AD modeling by Aβ peptides. On cultures of rat cortical neurons, PRE-084, T-817MA, and afobazole prevented cell death induced by Aβ_25–35_ [173,174] or Aβ_1–42_ [175]. NE-100 blocked the action of PRE-084 [173]. Pridopidine and 3-PPP reversed Aβ_1–42_-induced loss of mushroom spines in mice hippocampal cultures and impairment of LTP in acute hippocampal slices [176]. Afobazole inhibited the migration of microglia elicited by Aβ_25–35_ in vitro. The effect of afobazole was attenuated by Sigma1R antagonist BD-1047 [177]. Fluvoxamine, which has Sigma1R agonist properties, reduced γ-secretase-mediated cleavage of APP and Aβ secretion in CHO cells stably expressing human APP695 [157]. In mice, (+)-pentazocine, PRE-084, SA4503, T-817MA, donepezil, ANAVEX2-73, and imipramine demonstrated neuroprotective activity after ICV injection of Aβ_25–35_ [174,178,179,180,181,182], Aβ_1–42_ [167,168,183] or Aβ_1–40_ [184,185]. In the AD model induced by ICV injection of Aβ_25–35_, Sigma1R positive modulator OZP002, which has no ligand properties to Sigma1R but promotes chaperone activation, exhibited neuroprotective activity [186]. Sigma1R antagonists interfered with the neuroprotective activity of PRE-084, donepezil, ANAVEX2-73 [178,181,187], and OZP002 [186]. The contribution of Sigma1R activation to neuroprotective effects under ICV administration of Aβ_25–35_ to mice was clearly demonstrated by T. Maurice [188]. Subeffective doses of co-injected compounds with Sigma1R agonist properties caused neuroprotective effects. However, co-administration of subeffective doses of Sigma1R ligands and memantine had no protective effect [188] (Table S1).

Thus, despite contradictory data, most studies still indicate a decrease in the level of Sigma1R in the brain of experimental animals when AD is simulated by the administration of Aβ peptides (Figure 1). The disorders induced by Aβ peptides are neutralized by ligand activation of Sigma1R.

#### 3.1.3. Sigma1R and the Effects of the Chaperone Regulation in Transgenic Models of AD

Current information on genetic models of AD is presented in the detailed review by R. Sanchez-Varo et al. [189]. Neuro2a cells carrying Swedish mutant APP (APP_Swe_, p.Lys670_Met671delinsAsnLeu rs281865161) were characterized by elevated levels of Sigma1R and IRE1α in the MAM fraction [190]. As mentioned above, it is possible that upregulation of Sigma1R contributes to the stabilization of IRE1α and prevents proteasomal degradation of IRE1α under ER stress [108].

The results of in vitro experiments are consistent with in vivo studies. Transgenic mouse model AD with overexpression of the Swedish and London (APP_Lon_, p.Val717Ile rs63750264) mutant APP (APP_Swe/Lon_) is characterized by the formation of a large number of amyloid plaques [191]. Assessment of Sigma1R protein content in brain compartments of APP_Swe/Lon_ mice revealed an increase in Sigma1R level depending on their age and development of amyloid plaques. In the cortex, Sigma1R content increased by 10 months of age, which corresponds to severe Aβ pathology in this model. In the hippocampus, Sigma1R levels increased after 2 and 6 months of age, i.e., before Aβ deposition and at mild Aβ pathology, respectively. Interestingly, the Sigma1R mRNA level in the hippocampus of transgenic mice did not differ from WT animals by the age of 6 months, which may indicate stabilization and/or reduced degradation of Sigma1R. In the cerebellum, the amount of Sigma1R increased at 2 and 10 months of age, while staying unchanged in six-month-old animals. In mice carrying APP_Arc_ (rs63751039 p.Glu693Gly) mutation with mainly diffuse Aβ deposits in the subiculum area [192], Sigma1R levels did not change by 12 months the of age [161].

The dependence of Sigma1R cellular content on the degree of p-Tau aggregation found in the brain of AD patients was confirmed in transgenic pR5 mice, overexpressing the longest human Tau isoform with the p.Pro301Leu mutation (rs63751273). Sigma1R levels generally increased in CA1-subicular neurons but decreased in neurons with granulovacuolar degeneration [164].

Most of the data presented in this section indicate an increase in Sigma1R levels under the expression of FAD-inducing mutant proteins in in vitro and in vivo conditions (Figure 1). Sigma1R ligands with agonist activity pridopidine [176], T-817 MA [193], ANAVEX 3-71 [194,195], fluvoxamine [157] and Sigma1R positive modulator OZP002 [186] showed neuroprotective properties in transgenic AD models, similarly to AD models induced by the administration of Aβ peptides (Table S1).

### 3.2. BiP Chaperone in the Pathogenesis of Alzheimer’s Disease

#### 3.2.1. Relation of BiP to AD Pathogenesis in Clinical Trials

It was shown that SNPs in the promoter region of *HSPA5* (rs391957, rs17840761, rs3216733), affecting BiP chaperone expression, were associated with susceptibility to AD but not PD in Taiwanese populations [196,197]. It is interesting to note that the haplotype with low basal promoter activity but capable of enhancing *HSPA5* mRNA synthesis under ER stress conditions induced by thapsigargin, reduced the risk of AD [196]. These data are consistent with upregulation of BiP detected only in cytologically normal neurons of the hippocampus or entorhinal cortex of AD patients [198]. Published data indicate an elevated *HSPA5* expression on the temporal cortex, frontal cortex and hippocampus of AD patients [199,200,201,202]. BiP levels increase along with severity of AD, expressed in Braak score for neurofibrillary changes and amyloid deposits [201,202]. In hippocampal tissue homogenates and subicular neurons of postmortem brains of AD patients BiP and HSP70 content increased, however, similarly to Sigma1R, BiP level decreased in neurons with granulovacuolar degeneration. The content of BiP and Sigma1R chaperones changed unidirectionally depending on the studied brain region [164]. Downregulation of BiP is found in the temporal cortex of patients with SAD and, more pronounced, in FAD caused by mutations in *PSEN1* [94]. BiP level decreased in the parietal cortex but did not change in the cingulate gyrus, prefrontal cortex, and temporal cortex [203]. A number of studies also showed no significant differences in the mRNA or BiP protein content in brain samples of AD patients [101,204,205,206,207,208,209]. The results indicating the induction of BiP expression are consistent with the activation of IRE1α and PERK signaling of the UPR in the hippocampus and cortical areas of AD patients [199,207,210,211,212,213] (Figure 1).

#### 3.2.2. BiP Expression in AD Modeling with Aβ Peptides

In in vitro experiments, a decrease in cell viability is accompanied by an increase in the BiP level after exposure of cell cultures to Aβ_25–35_ [166,214,215,216,217,218,219,220,221], Aβ_1–40_ and Aβ_1–42_ peptides [92,93,222,223,224,225,226,227], which corresponds to the effects of ER stress inducers tunicamycin and thapsigargin [91,92]. In a number of studies, various compounds with cytoprotective activity exhibited the opposite effect, reducing BiP levels [93,215,217,218,219,220,227]. Despite the prevalence of experimental data, indicating an increase in BiP levels under Aβ peptides exposure, some studies showed no such change after incubation of SK-N-SH or HEK293 cells with oligomeric and fibrillar Aβ_1–42_ [228] and a decrease in BiP levels after exposure of Neuro2a cells to Aβ_25–35_ [229].

The results of in vitro experiments are consistent with in vivo damaging effects of Aβ_1–42_. Aβ_1–42,_ bilaterally microinjected into the entorhinal cortex of rats, impaired passive avoidance learning and memory as well as novel object recognition performance, which were accompanied by an increase in BiP and CHOP levels [230,231]. Intrahippocampal Aβ_1–42_ injection to rats caused an increase in BiP levels in the hippocampus on the third day of the experiment, which was combined with an increase in CHOP and cleaved caspase-12 [232]. ICV administration of Aβ_1–42_ to rats after 7 days also increased BiP content in the hippocampus and cerebral cortex [233]. The increase in BiP level induced by Aβ peptides is consistent with the UPR signaling activation in vitro [93,214,216,217,220,222,227,234,235].

#### 3.2.3. BiP Expression in Transgenic Models of AD

AD causative mutations in *PSEN1* (p.Ala246Glu rs63750526, exon 9 deletion (ΔE9) p.Ser290Cys;Thr291_Ser319del rs63750219) result in decreased *HSPA5* mRNA or BiP protein levels after incubation of transfected SK-N-SH neuroblastoma cells with tunicamycin. Similarly, BiP expression was reduced upon ER stress activation in HEK293 cells expressing other mutant variants of human *PSEN1* (p.Met146Val rs63750306, p.Ile213Thr rs63751309) [94,236]. Neuro2a cells expressing APP_Swe_ were characterized by reduced BiP expression in whole-cell extracts and microsomes [190]. In experiments on primary mouse cortical neurons *PSEN1* p.Ala246Glu knock-in mutation did not affect *Hspa5* mRNA levels in tunicamycin or thapsigargin induced ER stress models [95]. Similar data were obtained when ER stress was modeled in fibroblast culture from patients with FAD caused by other *PSEN1* mutations (p.Leu392Val rs63751416, p.Cys92Ser, p.Arg278Lys rs63751141) [96] and fibroblasts from *PSEN1* p.Ile213Leu (rs63750861) knock-in mice [236]. Thus, FAD mutations in *PSEN1* prevent BiP chaperone expression increase, which is consistent with ER stress inducers (tunicamycin, thapsigargin) or Aβ peptides action. It is possible that the detected ability of fibroblasts derived from FAD patient with mutant *PSEN1* p.Arg278Lys to increase Aβ_42_ secretion [96] may be related to insufficient functional activity of BiP [237,238]. The lack of BiP induction is consistent with the attenuation of UPR by mutant *PSEN1* variants. Thus, transfection of mutant *PSEN1* (p.Ile213Thr rs63751309 or ΔE9) caused decreased levels of p-IRE1, PERK, PERK-mediated eIF2α phosphorylation, activated ATF6p50, and impaired translocation of ATF6p50 to the nucleus [94,236,239].

Most studies on transgenic models of AD using mutant APP revealed an upregulation of BiP in the brain of experimental animals, which is consistent with the results of clinical studies and the effects of Aβ peptides. Elevated BiP content was found in hippocampal homogenates of mice expressing mutant APP (p.Glu693del) [240]. APP/PS1 (APP_Swe_/PS1 ΔE9) transgenic mice aged 6, 7 and 9 months were characterized by enhanced BiP expression in the hippocampus [241,242], cerebral cortex [226] or parietal cortex [242]. Administration of compounds with neuroprotective activity had the opposite effect, reducing the BiP level [242]. 3xTg mice expressing APP_Swe_, PS1 p.Met146Val, and Tau p.Pro301Leu demonstrated increased BiP levels in the hippocampus 8 or 12 months after birth [200,222,243]. Flavonoid luteolin with antioxidant properties in this AD model had neuroprotective activity, which was combined with decreased BiP levels in brain tissues [243].

In vivo AD model with 5XFAD mice overexpressing the five familial-inherited AD mutations (APP_Swe_, APP_Flo_ rs63750399, APP_Lon_, PS1 p.Met146Val, PS1 p.Leu286Val rs63751235) revealed biphasic changes in BiP protein levels. By the second month of animal life, the BiP level in cortical tissues increased and then gradually decreased to control values. Downregulation of BiP was accompanied by an increase in the content of cleaved caspase-12 and loss of neurons in the frontal cortex [244]. Likewise, BiP levels in brain homogenates from 5XFAD mice aged 4, 6, and 9 months did not differ, while the content of APP and PS1 significantly increased by the fourth month of the animals’ life [245]. Similarly to experiments on 5XFAD mice, BiP levels increased in brain homogenates of 5XFAD rats 2 months after the beginning of the experiment, which coincided with an increase in caspase-3 content [246]. These studies may indicate a relatively rapid depletion of neuronal ability to express BiP under conditions of overexpressing 5XFAD transgene, compared to less severe transgenic AD models. In the research of S. Hashimoto on transgenic mice of various ages, ex-pressing APP_Swe_, APP_Ibe_ (p.Ile716Phe), APP_Arc_ or Tau p.Pro301Leu no changes in BiP levels and other markers of ER stress in the hippocampus and cortex starting from three months were shown [247] (Figure 1). Changes in BiP expression induced by mutant variants of APP are consistent with the activation of UPR signaling in the early stages of experimental AD modeling [212,213,242,246] and the possibility of UPR depletion during the later stages [245,247].

### 3.3. Sigma1R and BiP Chaperones in the Pathogenesis of Alzheimer’s Disease, Summary

Thus, most studies indicate a decrease in the content of MAM-resident chaperone Sigma1R and its ability to bind ligands in the brain of AD patients depending on the severity of the disorders. These data are consistent with a decrease in Sigma1R levels in the brain of experimental animals depending on the time passed after ICV administration of Aβ peptides. It should be noted that the available scientific data do not record a decrease in Sigma1R levels in response to the expression of mutant forms of APP in in vivo AD models. It is possible that such results are caused by a relationship of Sigma1R activity and expression with APP metabolism in MAM [124] (Figure 1). In the vast majority of studies, the neuroprotective activity was detected for Sigma1R agonists, while Sigma1R antagonists inhibit their action (Table S1). Separate publications describe the protective properties of the Sigma1R antagonist NE-100.

The data presented in this section indicate an increase in BiP levels in the brains of AD patients depending on the severity of the disease. The increase in BiP level in cytologically normal neurons of AD patients, as well as in AD modeling by Aβ peptides administration and in transgenic in vivo AD models can be considered as manifestations of an adaptive cellular response aimed at restoring protein folding, which can be depleted as the disease progresses. However, similar to Sigma1R chaperone content fluctuations, BiP levels decrease as cellular damage intensifies. Reduced BiP levels in the brain of FAD patients with mutations in *PSEN1*, the demonstrated inability of cells carrying mutant *PSEN1* or *APP* to increase BiP expression and activate UPR signaling in response to ER stress inducers, and the relatively rapid decrease in BiP levels in 5XFAD transgenic animals may indicate a compromised response to ER stress and explain the early onset of FAD. Given the marker value of elevated BiP level for ER stress, the multidirectional changes in Sigma1R and BiP content in the brain of AD patients and in AD models in vitro and in vivo indicate a contribution of Sigma1R to ER stress attenuation (Figure 1).

## 4. Parkinson’s Disease

The main symptoms of PD including rigidity, akinesia or bradykinesia, and resting tremor are caused by loss of dopaminergic neurons and dopamine depletion in the basal ganglia, mainly in the substantia nigra pars compacta (SNc) [248]. Motor symptoms can be accompanied by depression and cognitive impairment. At a cellular level, markers of the disease are insoluble α-Synuclein (α-Syn) fibrils, which form Lewy bodies together with lipids. Depending on the disease progression (Braak stages), α-Syn inclusions are found in cholinergic and monoaminergic lower brainstem neurons in asymptomatic cases, basal forebrain in those with motor PD symptoms, and later in limbic and neocortical brain regions. Sporadic PD accounts for about 85% of all PD cases. The remaining cases are familial PD (FPD) with autosomal dominant or autosomal recessive inheritance. Most cases of early-onset FPD happen due to mutations in the α-Syn (*SNCA,* phenotype MIM 168601, 605543), parkin RBR E3 ubiquitin protein ligase (*PRKN*, phenotype MIM 600116), Parkinsonism associated deglycase (DJ-1 *PARK7*, phenotype MIM 606324), PTEN induced kinase 1 (*PINK1*, phenotype MIM 605909), leucine rich repeat kinase 2 (*LRRK2*, phenotype MIM 607060) genes [249,250,251].

### 4.1. Sigma1R Chaperone in the Pathogenesis of Parkinson’s Disease

#### 4.1.1. Relation of Sigma1R to PD Pathogenesis in Clinical Trials

It was shown that Sigma1R agonist [^11^C]SA4503 binding was significantly lower on the more affected side of the anterior putamen compared to the less affected side in PD patients. No differences were found in the volume of distribution of [^11^C]SA4503 in different brain structures of early drug-naive PD patients compared to healthy volunteers [252,253].

#### 4.1.2. Relation of *Sigmar1* Gene Downregulation to the Development of Parkinsonism

*Sigmar1^−/−^* mice reproduce a phenotype close to PD. Two months old *Sigmar1^−/−^* animals showed impaired performance on the rotarod [126,254]. By the age of 6 months *Sigmar1^−/−^* mice demonstrate decreased number of TH+ cells in the SNc, impairment of movement coordination, accumulation of monomers and oligomers of phosphorylated α-Syn, as well as inhibition of proteosomal degradation in neurons [255].

*SIGMAR1* knockdown promotes MPP^+^-induced reduction in SH-SY5Y cell viability [256]. It is interesting that in the previously published study, MPTP administration to *Sigmar1^−/−^* and *Sigmar1^+/−^* mice starting at 3 months of age did not cause PD-specific abnormalities [257]. These data are consistent with a decrease in Aβ_25–35_-induced hippocampal neuronal cell death and spatial cognitive deficits in *Sigmar1^+/−^* mice [171].

Auto-oxidation of dopamine (DA) promotes neuronal damage and is involved in the pathogenesis of PD [258,259,260]. Treatment of CHO cells with DA at a concentration close to physiological (10 μM) within 1 h induced an increase in Sigma1R expression, which appeared to be dependent on ROS generation and was eliminated by ascorbic acid. In *Sigmar1* knockdown CHO cells, DA treatment enhanced ROS production, decreased Bcl-2 levels, and induced apoptosis compared to WT cells. However, the protective effect of Sigma1R on cells appeared to be unrelated to the UPR activation, which may be due to enhanced chaperone expression by DA [261].

#### 4.1.3. Sigma1R Expression and Effects of Its Ligands in PD Models

Reduction of ER-mitochondria associations in SH-SY5Y cells expressing mutant α-Syn was not accompanied by changes in Sigma1R content [262]. However, it was shown on *PARK7* knockout M17 cells that disruption of the IP_3_R3-GRP75-VDAC1 complex and accumulation of IP_3_R3 in MAM is accompanied by a decrease in MAM Sigma1R levels [263].

When PD was simulated in mice by administration of 6-OHDA, a slight increase in the total number of Sigma1R immunoreactive cells in the ipsilateral striatum was revealed. The Sigma1R agonist PRE-084 in this PD model restored motor activity, increased the number of TH+ neurons in the SNc and DA levels in the striatum. PRE-084 did not affect Sigma1R expression but altered Sigma1R redistribution in nigral dopaminergic neurons and astrocytes [264], which is consistent with Sigma1R activation [136]. Pridopidine, a non-selective Sigma1R ligand with agonist properties had a similar restorative effect on the behavior and number of TH+ neurons in the SNc [265]. PRE-084 and pridopidine did not cause neuroprotective effects in *Sigmar1* knockout mice [264,265]. The antidepressants fluvoxamine, fluoxetine, and imipramine are known to have Sigma1R agonist properties [266,267]. SSRI fluoxetine improved spatial cognitive deficits in rats with unilateral 6-OHDA injection [268]. The anxiolytic afobazole, which agonistically affects Sigma1R [269] exhibited neuroprotective properties in the 6-OHDA model of PD in mice, restoring motor activity of experimental animals [270,271], DA levels in the striatum [270,271,272] and increasing the number of TH+ neurons in the SNc [270]. The effects of afobazole were significantly attenuated by the Sigma1R antagonist BD-1047 [270,271] (Table S1).

Sigma1R levels in the SNc of C57Bl/6 mice with MPTP-induced parkinsonism also did not change. As in the PD model induced by administration of 6-OHDA, PRE-084 exhibited neuroprotective properties, restoring motor ability and mitophagy in mice with MPTP-induced parkinsonism. PRE-084 had cytoprotective activity in vitro, attenuating MPP^+^-induced death of SH-SY5Y cells [256]. Fluoxetine and imipramine eliminated motor deficits, increased DA levels and prevented loss of TH+ fibers in striatum of mice treated with MPTP [273,274] (Table S1).

In contrast to the results of clinical studies and experiments on rodents, PD modeling in rotenone-treated zebrafish revealed elevated levels of *sigmar1* mRNA and Sigma1R protein in the brain. This effect was combined with an increase in *vdac* and *pink1* gene expression and a decrease in the level of mitochondrial Ca^2+^ [275]. At the same time, imipramin attenuated rotenone-induced alterations of rat motor activity, restored the content of striatal monoamines and increased the number of TH+ neurons in the SN [276] (Table S1).

**Figure 2:**
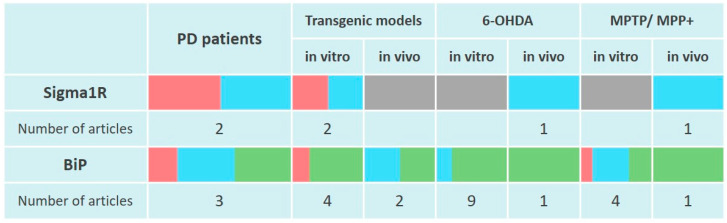
Changes in Sigma1R and BiP chaperones expression in the pathogenesis of Parkinson’s disease. The table reflects experimentally recorded changes in the expression of Sigma1R and BiP chaperones, denoted in relative units, according to Section 4.1 and Section 4.2. PET data were used to visualize Sigma1R content in the brains of PD patients. To visualize mRNA or protein levels following markers were used: green—content increased, blue—content did not change, red—content decreased, grey—not enough data.

Scientific data presented in the section indicate the absence of induction of Sigma1R expression in PD modeling by neurotoxins in vitro and in rodent models (Figure 2). At the same time, Sigma1R ligands with agonist activity demonstrate neuroprotective properties in vivo.

### 4.2. BiP Chaperone in the Pathogenesis of Parkinson’s Disease

#### 4.2.1. Relation of BiP to PD Pathogenesis in Clinical Trials

BiP protein levels decreased in post-mortem human brain samples from the SNc and hippocampus but did not change in the cortex of sporadic PD patients. A decrease in BiP content corresponded the ATF4 downregulation in the SNc [208]. Elevated mRNA expression of *HSPA5* was detected in caudate, prefrontal cortex, cingulate gyrus, and parietal cortex. However, in this study, BiP protein levels did not change, or, on the contrary, decreased in the temporal cortex and cingulate gyrus [277]. It is possible that the reduced level of BiP may be associated with a relatively short half-life of the chaperone due to its enhanced degradation [278]. BiP expression features were revealed in patients with PD and dementia with Lewy bodies (DLB), which were characterized by elevated BiP content in cingulate gyrus but not in parietal, prefrontal or temporal cortex regions compared to the control group [203,277] (Figure 2). These data are consistent with the detection of p-PERK immunoreactivity only in α-Syn positive neurons from post-mortem PD brains [279].

#### 4.2.2. BiP Expression in Genetic Models of PD

Similar to AD, the risk of developing PD increases with aging. In 24-month-old Wistar rats compared to 2-month-old animals, α-Syn levels increased contrary to BiP, with no significant decline in SNc TH levels. The recorded changes may reflect the initial links in the pathogenesis of PD. In addition, SNc cells of old animals did not respond with BiP upregulation to human α-Syn overexpression [280].

The ability of BiP to form a complex with α-Syn was shown in vitro using immunoprecipitation [281,282]. Exposure of neurons to exogenous α-Syn increased the BiP levels in specific microdomains of the plasma membrane [282], which is consistent with higher BiP content in the cell surface area under ER stress, even taking into account the increase in total BiP level [283]. Higher BiP level was also found in HEK293 cells carrying truncated α-Syn (1–120) and in the brain of α-Syn (1–120) transgenic mice [281]. Similarly, BiP levels increased in the spinal cord and brain stem of mice with overexpression of human mutant α-Syn [284] (Figure 2). Elevated BiP expression is consistent with the activation of PERK and ATF6 signaling cascades of UPR under conditions of human WT α-Syn overexpression in rat SNc [285].

Induction of BiP was significantly attenuated in *Park7* knockout mouse cortical neurons and *PARK7* knockdown SH-SY5Y cells, which was combined with a decrease in ATF4 expression [105] (Figure 2).

#### 4.2.3. BiP Expression in 6-OHDA Models of PD

Weakening of *Hspa5* gene activity did not affect the phenotypic manifestations of 6-OHDA-induced parkinsonism. Thus, behavioral disorders and biochemical abnormalities in the SNc of *Hspa5*^+/−^ mice modeled by unilateral 6-OHDA lesion did not differ from those found in WT animals [286].

Cultured sympathetic neurons from newborn mice reacted by increasing BiP level 8 h after treatment with low concentrations of 6-OHDA (3 and 5 μM) [287]. 6-OHDA at a 10 μM concentration did not increase BiP levels in the Mes23.5 cell line after 4 h but caused PERK activation [288]. Treatment of cultured rat cerebellar granule neurons with 6-OHDA at a higher concentration (50 µM) raised BiP levels after 2 and 6 h [289].

Exposure of PC12 cells to 6-OHDA (80–100 μM) for up to 24 h caused an increase in the expression of CHOP and UPR signaling proteins as well as BiP after 8 h of incubation with the neurotoxin [287,290]. The neuroprotective effect of the compound with antioxidant properties studied in this work was associated with lower BiP levels [290]. The mRNA level of *Hspa5* was found to increase in MN9D cells treated with 6-OHDA but not in MPP^+^, where *Hspa5* expression was slightly decreased. Changes in BiP protein levels showed a slight increase over 12 h with 6-OHDA treatment but not with MPP^+^, which was consistent with mRNA levels. However, in the same study, BiP content did not change in primary mesencephalic cultures following 6-OHDA treatment over 12 h [291]. A number of studies have shown that exposure of dopaminergic SH-SY5Y cells to 6-OHDA (25 and 50 µM) for 2 to 12 h causes an increase in BiP expression [289,292,293]. Similar in the mechanism of cell penetration and activation of ROS production to 6-OHDA, paraquat [294] also caused an increase in BiP levels after 48 h of incubation with SH-SY5Y cells [295]. In experiments on other dopaminergic cell lines SN4741 or MN9D 6-OHDA (10 µM or 150 µM) raised BiP expression after 4 or 9 h, similar to the effects of tunicamycin or thapsigargin [99,296]. The cytoprotective effect of the cGMP and ER stress inhibitor 4-phenylbutyric acid (4-PBA) on SN4741 cells was accompanied by a decrease in BiP levels [296]. Plant glycoside salidroside which possesses antioxidant properties [296], decreases BiP expression in a similar way, weakening iNOS and nNOS expression [297]. Thus, in vitro studies indicate an increase in BiP expression, which is consistent with the activation of UPR signaling [99,287,288,289,291,293,296].

The ability of 6-OHDA to cause an upregulation of BiP expression was confirmed in vivo after ICV injection of the neurotoxin. After 7 days, the level of caspase-12 and CHOP increased, along with BiP in the striatum of ICR mice, and caspase-3 in the SNc [298]. The enhancement of BiP expression in nigrostriatal neurons, detected 14 days after 6-OHDA injection in rats’ SNc, was combined with an increase in ATF4 co-staining, decrease in DAT and TH levels, and higher α-Syn expression. The neuroprotective effect of phenylethanoid glycoside echinacoside, which exhibits antioxidant properties, was counteracted by lower expression of these proteins [299].

Analysis of current data indicates an increase in BiP content in neurons and cell cultures when PD was modeled by 6-OHDA administration (Figure 2).

#### 4.2.4. BiP Expression in MPTP and MPP^+^ Models of PD

Twelve hours after primary mesencephalic culture MPP^+^ treatment, the BiP level did not change [291]. Experiments on human teratocarcinoma NT2 cells show that MPP^+^, the active metabolite of MPTP, can activate UPR in an early phase, increasing BiP levels 2 h after treatment. A decrease in mitochondrial membrane potential (Δ*Ψ*m), ER Ca^2+^ content, mitochondrial Ca^2+^ uptake, and enhanced caspases activation along with a decrease in BiP content are most significant after 24 h [300]. Higher BiP levels were also recorded in PC12 cells 24 h after MPP^+^ treatment [301]. An insignificant increase in BiP content was observed in MN9D cells 24 h after the 150 μM MPP^+^ treatment [99]. However, as the period after SH-SY5Y MPP^+^ treatment was prolonged up to 48 h, a decrease in BiP and an increase in CHOP levels were observed. Rutin protected cells and caused an inverse effect on these indicators [302]. BiP expression increase during early stages of MPP^+^ administration is consistent with the activation of all three UPR branches within 8 h after toxin addition [99,287,291,301].

Similar to the effect of 6-OHDA, in vivo PD modeling by MPTP injection causes upregulation of BiP in the brain of experimental animals (Figure 2). Thus, the damaging effect of chronic administration of MPTP with probenecid on TH+ neurons of mice was accompanied by elevated BiP expression. Moreover, BiP level increased in GFAP-positive activated astrocytes, but not in Iba1-positive microglia [303]. The impaired motor activity of mice 48 h after the MPTP course was accompanied by an increase in the BiP, IRE1α and CHOP content. The neuroprotective effect of δ opioid receptor agonist but not L-DOPA reduced these parameters [304]. Impaired motor activity and decreased number of TH+ neurons in the SNc, detected 8 days after five-day MPTP administration to C57Bl/6 mice, were accompanied by elevated BiP levels in brain homogenate combined with the activation of IRE1α signaling [305,306]. The neuroprotective effect of apelin-13 or apelin-36 neuropeptides was combined with a decrease in BiP levels [305,306].

#### 4.2.5. BiP Expression in Rotenone Models of PD

Twenty-four hours after treatment of MN9D cells with 200 μM mitochondrial complex I inhibitor rotenone [307], BiP content decreased along with a reduction in cell viability [99]. After 24 h rotenone-treated SH-SY5Y cells showed no change in BiP expression, while caspase-3 activation intensified and cell viability dropped. Despite the lack of data on the induction of BiP expression by rotenone, the toxin was shown to activate IRE1α and PERK signaling in vitro [287].

In in vivo experiments 4 weeks after rotenone injection into the SNc of C57Bl/6 mice, the mRNA levels of *Hspa5 (BiP), Atf4*, *Atf6* and *Xbp1* genes were increased. The neuroprotective effect of metformin was observed along with decreased BiP expression [308].

### 4.3. Sigma1R and BiP Chaperones in the Pathogenesis of Parkinson’s Disease, Summary

Thus, there is little or no induction of Sigma1R chaperone function in the brain of PD patients and in PD models according to current data (Figure 2). Several studies have convincingly demonstrated the possibility of achieving neuroprotective effects in PD models due to ligand activation of Sigma1R (Table S1).

Upregulation of *HSPA5* transcription along with lower BiP protein level in the brain are typical for PD. This peculiarity is consistent with the pattern of BiP expression in MPP^+^ and rotenone models of PD that are based on primary mitochondrial damage [294,309]. An increase in BiP expression and activation of UPR signaling proteins was a trait of 6-OHDA and MPTP models of PD, which may indicate the UPR induction by oxidative stress [294] (Figure 2).

## 5. Amyotrophic Lateral Sclerosis

Amyotrophic lateral sclerosis (ALS) is a neurodegenerative disease caused by loss of motor neurons in the brain and spinal cord. It is manifested by muscle weakness of the limbs (spinal symptoms) and/or dysarthria and dysphagia (bulbar symptoms). ALS is characterized by a steadily progressive course which, after 3–5 years, leads to the loss of the ability to stand or walk and overwhelming number of patients require mechanical assistance to breathe. About half of patients develop cognitive and/or behavioral disorders. About 90% of ALS cases are accompanied by pathological TDP-43 aggregation in human motor neurons [310,311]. In the European population, about 20% of ALS cases are caused by mutations in 24 genes, about 70% of which are caused by mutations in the four major genes. The largest number of familial ALS (FALS) cases is caused by a GGGGCC repeat in a noncoding region of C9orf72 (phenotype MIM 105550). Other relatively common causes of autosomal dominant ALS are mutations in the superoxide dismutase 1 (*SOD1*, phenotype MIM 105400), TAR DNA-binding protein 43 (TDP-43 *TARDBP*, phenotype MIM 612069), and RNA-binding protein FUS (*FUS*, phenotype MIM 608030) genes. Mutant ER-resident proteins Sigma1R, (*SIGMAR1*, phenotype MIM 614373) and vesicle-associated membrane protein-associated protein B/C (*VAPB*, phenotype MIM 608627) also cause ALS [312,313,314].

### 5.1. Sigma1R Chaperone in the Pathogenesis of Amyotrophic Lateral Sclerosis

#### 5.1.1. Sigma1R Expression in Motoneurons of Patients with ALS

**Figure 3:**
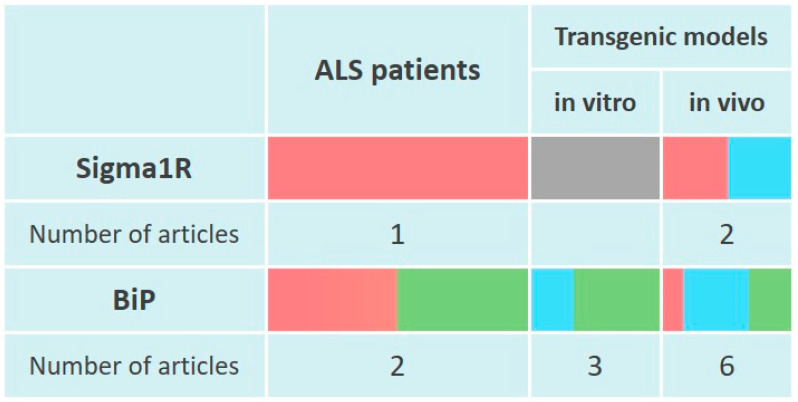
Changes of Sigma1R and BiP chaperones expression in the pathogenesis of amyotrophic lateral sclerosis. The table reflects experimentally recorded changes in the expression of Sigma1R and BiP chaperones, denoted in relative units, according to Section 5.1 and Section 5.2. To visualize mRNA or protein levels following markers were used: green—content increased, blue—content did not change, red—content decreased, grey—not enough data.

Alpha-motoneurons of preferentially sporadic ALS patients are characterized by an abnormal redistribution of Sigma1R and formation of ubiquitinated aggregates that contribute to the UPR. In addition, reduced Sigma1R levels have been shown in lumbar spinal cord specimens of ALS patients [135] (Figure 3).

#### 5.1.2. Motor Neuron Dysfunction Caused by Inactivation of the *Sigmar1* Gene

Knockdown of *Sigmar1* in NSC-34 motor neuron-like cells after 48 h causes an increase in BiP levels and activation of the PERK branch of the UPR, which are further enhanced by thapsigargin. In addition, cell death is promoted by a complex of disorders including changes in ER morphology, an increase in intracellular Ca^2+^, alteration of the mitochondrial membrane potential, caspase-3 activation, and cytochrome C release [135].

Similarly, activation of the PERK-dependent UPR pathway was shown in experiments where neuromuscular dysfunction in 2.5- and 5-month-old *Sigmar1^−/−^* mice was combined with an increase in p-eIF2α levels in spinal motor neurons [126]. In cultures of motor neurons derived from *Sigmar1^−/−^* mouse embryos, the number of IP_3_R3-VDAC1 interactions decreased. A less pronounced but significant decrease in IP_3_R3-VDAC1 interactions was observed after exposure to Sigma1R antagonist NE-100. Electron microscopy revealed a 1.6-fold decrease in the total number of mitochondria opposed to the ER in the motor neurons of 2.5-month-old mice [126]. Another study showed a similar decrease in MERC areas in *Sigmar1^−/−^* mice motor neurons along with elimination of IP_3_R3 [127].

*Sigmar1^−/−^* animals exhibit phenotypic traits typical to ALS [315]. The firing frequency of *Sigmar1^−/−^* juvenile mice motoneurons was significantly higher than that of WT mice, which may be related to differences in the activity of potassium channels. After 3 months of life, *Sigmar1^−/−^* mice lose weight faster, demonstrate earlier decline in swimming performance, and lower life expectancy [316]. Between 2.5 and 5 months of life, *Sigmar1^−/−^* mice show a progressive decrease in the innervation of neuromuscular junctions. By 5 months of age, motor neuron degeneration and lower muscle strength are detected. Primary motor neurons extracted from *Sigmar1^−/−^* mouse embryos showed a significant reduction in axonal length compared to WT neurons [126]. In the context of studying the contribution of Sigma1R to the pathogenesis of ALS, a decrease in latency to fall in the rotarod test was found in *Sigmar1^−/−^* at 12 but not 5 months of age [127], which is consistent with the results of other studies [126,254].

#### 5.1.3. Disorders Induced by ALS-Causative Mutations in the *SIGMAR1* Gene

Several causative mutations in the *SIGMAR1* gene are known for autosomal recessive juvenile FALS (ALS16), of which p.E102Q and p.Leu95Profs are the most functionally studied. For a review, see [138]. The p.E102Q mutation has been identified in the consanguineous family from Saudi Arabia [317]. It has been shown that p.E102 is required for the integrity of the C-terminal ligand-binding domain [318]. The p.E102Q mutation contributes to higher-order Sigma1R oligomers degradation [318,319]. On the other hand, p.E102Q Sigma1R is capable of forming aggregates that accumulate in cellular compartments, causing structural alterations of the ER, nuclear envelope and mitochondria [320]. It is shown that impaired ability to form Sigma1R-IP_3_R3 complex in cells carrying p.E102Q Sigma1R is consistent with disturbances of IP_3_R-mediated mitochondrial Ca^2+^ mobilization and ATP production. Proteasome activity of these cells is disrupted, while autophagic cell death under ER stress conditions enhances [127,320,321]. In addition, mutant Sigma1R has an impaired ability to bind to BiP in response to increasing Ca^2+^ concentration, while interactions with NMDA NR1 subunit, μ opioid receptor, and histidine triad nucleotide-binding protein 1 (HINT1) increase [132]. Drosophila flies expressing p.E102Q Sigma1R can be considered as a model of ALS16. They are characterized by impaired motor activity, abnormal mitochondrial fragmentation and reduced ATP levels in neurons [322].

The c.283dupC mutation (p.Leu95Profs rs780136067), resulting from paternal uniparental disomy for chromosome 9, was found in a non-consanguineous Hispanic family [127]. In in vitro experiments, expression of p.Leu95Profs or p.E102Q caused increased Ca^2+^ flux into the cytoplasm and decreased mitochondrial Ca^2+^ flux in response to ATP addition compared to WT Sigma1R [127].

#### 5.1.4. Sigma1R Expression and Effects of Ligands of the Chaperone in ALS Models

A decrease in Sigma1R expression was detected in lumbar motoneu-rones from transgenic hSOD1 p.G93A (rs121912438) mice at 1 month of age [323]. However, in another study, Sigma1R expression in the lumbar spinal cord of transgenic mice with the p.G93A hSOD1 mutation (ALS1) did not differ from that of WT animals [324] (Figure 3).

PRE-084 preserves motoneurons and neuromuscular connections, reduces microglial immunoreactivity, improves spinal motoneuron function and locomotor performance, prolongs survival of the transgenic mice [324,325,326]. Similar to PRE-084, Sigma1R agonist SA4503 reduced microgliosis. Interestingly, the Sigma1R antagonist BD-1063 also enhanced nerve conduction and reduced spinal motoneurons death [326]. Pridopidine, which has Sigma1R agonist properties, eliminated gait abnormalities in hSOD1 p.G93A mice [327].

PRE-084 restored in vitro mitochondrial activity in an ALS model (ALS8) on differentiated NSC-34 cells carrying p.P56S *VAPB*. It was shown that transfected cells form aggregates consisting of VAPB and Sigma1R while soluble Sigma1R decreases. PRE-084 reduced mutant VAPB aggregation and facilitated the degradation of soluble mutant VAPB [135] (Table S1). Presented data indicate neuroprotective properties of Sigma1R ligands in in vitro and in vivo ALS models.

### 5.2. BiP Chaperone in the Pathogenesis of Amyotrophic Lateral Sclerosis

#### 5.2.1. BiP Expression in Cells of Patients with ALS

BiP positive motor neurons were more frequently observed in patients with sporadic ALS compared to controls [328]. *HSPA5* expression was also higher in the spinal cord derived from sporadic cases of ALS [135,199]. In an attempt to detect ALS biomarkers, PBMCs and fibroblasts from ALS patients were analyzed. ALS PBMCs were characterized by an increased content of BiP [329], whereas in ALS fibroblasts *HSPA5* expression did not reach statistical significance compared to the control group [330]. BiP level decreased in PBMCs from patients with early ALS compared to late ALS [331] (Figure 3). Elevated BiP expression is consistent with the activation of IRE1α [98,199,329] and PERK [98,332,333] signaling in post-mortem spinal cord samples from ALS patients.

#### 5.2.2. BiP Expression in Experimental Models of ALS

In COS7 cells carrying hSOD1 p.G93A, the formation of SOD1 aggregates colocalized with BiP was combined with an increase in BiP expression [334]. However, transfection of hSOD1 p.G93A into NSC-34 motor neuron cells had no effect on BiP levels [335]. SH-SY5Y cells expressing hTDP-43 p.A315T (ALS10, TARDBP rs80356726) showed an increase in BiP level 72 h after transfection [336].

The hSOD1 p.G93A mutation was shown to be associated with SOD1 and BiP colocalization not only in vitro [334], but also in the lumbar spinal cord of SOD1 p.G93A transgenic mice. Moreover, BiP content was detected in all Lewy body-like hyaline inclusions (LBHIs), which suggests a close relationship between mutant SOD1 and BiP in these structures [337]. In contrast to hSOD1 p.G93A transgenic mice, LBHIs in sporadic ALS patients were not BiP positive, indicating differences in the involvement of ER stress in the pathogenesis of familiar and sporadic ALS [328].

In the spinal cord of hSOD1 p.G93A mice, BiP content did not change significantly at presymptomatic (4 or 12 weeks) and late symptomatic groups (20 weeks) [338] as well as in the pre-onset (63–69 days), onset (102–121 days), and end stage (135–152 days) groups [339]. Similarly to the previous data, BiP expression did not change in the dorsal root ganglia of hSOD1 p.G93A mice at the 126th day of life [340]. Other researchers also demonstrated the absence of changes in the spinal cord BiP level on the 150th day of life of transgenic mice in this experimental model. A decrease in BiP levels was recorded in the motor cortex of symptomatic hSOD1 p.G93A mice and in hSOD1 p.G93A primary microglia [335].

BiP was downregulated in the spinal cord of hSOD1 p.G93A mice reproducing the early ALS phenotype, similar to the PBMCs from patients with early ALS [331]. Interestingly, *Hspa5^+/−^* mice did not differ from WT animals in weight gain, neurological score, motor activity, and several histopathological markers in the ventral horn of the spinal cord. However, double mutant mice (hSOD1 p.G93A/*Hspa5*^+/*−*^) were characterized by pathological changes in these parameters compared to hSOD1 p.G93A and WT mice [286]. Together, the above results suggest the contribution of decreased BiP expression to the development of disorders typical to ALS. Only one study showed the upregulation of BiP in the spinal cord of 130 days old (18.5 weeks) transgenic hSOD1 p.G93A mice [341]. BiP levels also increased in skeletal muscle but not in cardiac muscle or liver tissue at postnatal days 70–140. The maximum increase in BiP level at the late stages of ALS modeling coincided with the increase in CHOP content [342]. Similarly, BiP expression was increased in spinal cord motoneurons of transgenic mice expressing mutant hSOD1 p.L84V (rs121912452) or p.H46R (rs121912443) [334] (Figure 3).

### 5.3. Sigma1R and BiP Chaperones in the Pathogenesis of Amyotrophic Lateral Sclerosis, Summary

Analysis of current data indicates a decrease in Sigma1R levels in spinal cord samples of ALS patients. The results of clinical studies are consistent with the development of an ALS-like phenotype with decreased activity of the *Sigmar1* gene in vivo and impaired mitochondrial function induced by ALS-causative *SIGMA1R* mutations in vitro (Figure 3). Ligand activation of Sigma1R chaperone function has a protective effect on motoneurons in ALS models (Table S1).

In ALS patients’ motoneurons and cell cultures carrying hSOD1 p.G93A an elevated BiP expression was observed, which, together with UPR signaling activation is consistent with the chronic ER stress development. However, studies on hSOD1 p.G93A transgenic model of ALS in vivo generally show no effect of the mutation on BiP expression or decrease in chaperone levels in spinal cord neurons in case of early onset or progression of ALS-like symptoms (Figure 3).

## 6. Huntington’s Disease

Huntington’s disease (HD) is an autosomal dominant neurodegenerative disorder caused by an increase in ≥36 CAG repeats in the *HTT* gene encoding the polyglutamine segment of the huntingtin protein (phenotype MIM 143100). Symptoms of the disease are caused by the loss of striatal medium spiny and cortical neurons. Clinically, the disease manifests as increasing involuntary choreiform movements, cognitive and behavioral difficulties, culminating in dementia. The accumulation of mutant HTT oligomers, which subsequently form fibrils and large inclusions in both the cytoplasm and nucleus is a cellular marker of HD. For a review, see [343].

### 6.1. Sigma1R Chaperone in the Pathogenesis of Huntington’s Disease

#### 6.1.1. Sigma1R Expression in HD Patients’ Brain and in Experimental Disease Models

**Figure 4:**
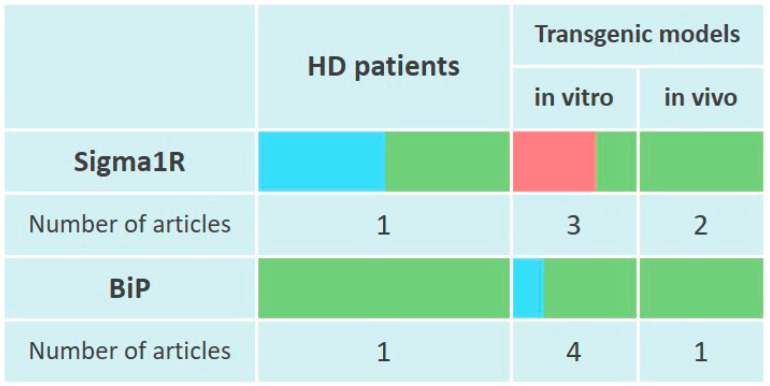
Changes of Sigma1R and BiP chaperones expression in the pathogenesis of Huntington’s disease. The table reflects experimentally recorded changes in the expression of Sigma1R and BiP chaperones, denoted in relative units, according to Section 6.1 and Section 6.2. To visualize mRNA or protein levels following markers were used: green—content increased, blue—content did not change, red—content decreased.

Elevated Sigma1R levels were found in striatal samples from patients with severe, but not moderate HD. These data are consistent with in vivo studies, which found a statistically significant increase in Sigma1R levels in YAC128 mice [344] striatum by 12 months of life [345]. However, in YAC128 mice primary striatal neuron culture Sigma1R levels appeared reduced [346] (Figure 4).

Expression of toxic mutant huntingtin (Htt96Q) oligomers in HEK 293 cells 24 h after transfection caused an approximately 2-fold increase in Sigma1R levels compared to cells expressing WT Htt20Q. Interestingly, this condition appeared to be unrelated to chaperone expression and might be a consequence of stabilization and reduced degradation of the protein [347]. In addition, Htt96Q reduced the colocalization of Sigma1R and BiP, which corresponds to the dissociation of the Sigma1R-BiP complex under ER stress [107,347]. On PC6.3 cells (a subclone of the PC12 cell line) carrying mutant huntingtin protein (Htt120Q) the level of Sigma1R was decreased 24 h after transfection compared to control and Htt18Q cells [348].

In an in vitro HD model based on the transfection of HeLa cells with GFP-labeled huntingtin bearing the Q74 repeat (HttQ74), Sigma1R incorporation into the majority of intranuclear mutant huntingtin aggregates was shown. Downregulation of *SIGMAR1* increased the number of mutant huntingtin aggregates in both the cytoplasm and nucleus. Similar results were obtained after application of epoxomicin, a specific proteasome inhibitor. Overexpression of *SIGMAR1* prevented huntingtin aggregate formation. These results indicate a link between Sigma1R and mutant Huntingtin degradation in the nucleus via ERAD [349] (Figure 4).

#### 6.1.2. Effects of Sigma1R Ligands in Experimental Models of HD

The protective effect of enhancing *SIGMAR1* expression is consistent with the action of Sigma1R agonist PRE-084. In cells expressing Htt120Q, the compound prevented ROS production and activation of caspases 3/7 and 12. This resulted in a reduction of cell death to less than half of the usual rate [348]. It was shown that the effect of PRE-084 was caused by increasing calpastatin and activation of NF-kB signaling [348], which is consistent with the decrease in calpain activity in the ALS model in vitro [127]. In a number of studies, pridopidine, which agonistically affects Sigma1R, exhibited neuroprotective properties in HD models. Pridopidine ameliorates ROS production and restores mitochondrial function in neurons, delays the onset of motor deficits of YAC128 mice [346]. The compound induces remodeling of mutant Htt in the stratum and improves motor functions of R6/2 mice. The in vitro antiapoptotic effect of pridopidine was blocked by the Sigma1R antagonist NE-100 [350]. In the recent work by M. Shenkman et al. Sigma1R-dependent mechanisms of pridopidine action were revealed in in vitro experiments [347]. Twenty-four hours after treatment of cells expressing Htt96Q, the drug prevented ER stress by reducing the levels of p-eIF2α, ATF6 cleaved fragment, and XBP-1s mRNA. These effects of pridopidine corresponded to an increase in Sigma1R levels, restoration of Sigma1R and BiP colocalization, increase in the sequestration of mutant Htt into insoluble aggregates [347]. Fluoxetine, which has Sigma1R agonist properties, enhanced neurogenesis in the dentate gyrus and improved cognitive functions in transgenic R6/1 mice [351] (Table S1).

### 6.2. BiP Chaperone in the Pathogenesis of Huntington’s Disease

In striatum samples from HD patients increased BiP and p-IRE1 levels was detected compared to the control group [103]. In vivo studies demonstrated an increase BiP levels in striatal crude homogenates of heterozygous Q175/Q7 knock-in HD mice after 5 months [352]. In the striatum of YAC128 HD mice aged 18 months, BiP and p-IRE1 levels were also elevated. At the same time, BiP content did not change in the cortex, hippocampus, and cerebellum [103], indicating ER stress activation in brain structures damaged by HD.

In HEK 293 cells expressing myc-tagged Htt96Q, the BiP level increased 16 h after transfection and positively correlated with the formation of Htt96Q oligomers and insoluble Htt aggregates. The elevated BiP levels corresponded to PERK signaling activation, which was observed 13 h after transfection, whereas ATF6 and IRE1α branches were activated at 24 and 30 h, respectively [353]. In Neuro2a cells carrying Htt150Q, BiP levels also increased 3-fold after 48 h compared to control and Htt20Q cells, reflecting the development of ER stress [354].

PC6.3 cells carrying N-terminal mutant Htt proteins showed the dependence of BiP expression on the length of polyQ repeats. Activation of caspase-3, increased cell death and protein aggregates formation detected in Htt39Q, Htt53Q and Htt120Q compared to Htt18Q and control cells 24 h after transfection, were accompanied by an elevated BiP expression, PERK-mediated phosphorylation of eIF2α and translocation of activated ATF6 to the nucleus [355] (Figure 4).

However, in the work of P. Lajoie et al. contrasting results were obtained [356]. Neuro2a cells carrying Htt73Q or Htt145Q showed no change in BiP levels 48 h after transfection compared to controls and cells expressing nonpathological Htt23Q. BiP induction also did not occur in mouse striatal cell lines expressing two knock-in copies of full-length mutant (STHdh Q111/111) Htt under the endogenous promoter compared to WT (STHdh Q7/7) cells (Figure 4). These cells exhibited significant decrease in BiP-GFP intracellular mobility compared to Q23-expressing cells. Exposure of control cells to the acute ER stress inducer tunicamycin had a similar effect. This effect was not observed in STHdh Q111/111, possibly due to the constitutive expression of mutant Htt at near endogenous levels and adaptation to low level stress. In addition, STHdh Q111/111 striatal cells appeared to be more sensitive to tunicamycin, which was expressed in lowering of BiP-GFP intracellular mobility already after 30 min and caspase-3 activation 16 h after tunicamycin addition [356].

### 6.3. Sigma1R and BiP Chaperones in the Pathogenesis of Huntington’s Disease, Summary

The above data allow us consider the enhancement of Sigma1R expression in the brain of HD patients and in vivo HD models to be an adaptive mechanism aimed at attenuating Htt aggregation. In HD models in vitro, the protective effect on cells develops under conditions of enhanced Sigma1R expression (Figure 4). These results are consistent with the neuroprotective activity of Sigma1R ligands possessing agonist properties (Table S1). Similarly, results of most studies indicate a compensatory role of elevated BiP levels in response to the induction of mutant Htt expression (Figure 4).

In the following sections, chaperone-dependent pharmacodynamic mechanisms of neuroprotection corresponding to the contribution of Sigma1R and BiP to the pathogenesis of neurodegenerative diseases will be analyzed.

## 7. Sigma1R-Dependent Neuroprotective Mechanisms Caused by UPR Signaling Regulation

### 7.1. Features of the Neuroprotective Action of Sigma1R Ligands

Data presented in the review indicate the contribution of the MAM chaperone system and UPR signaling cascades to the pathogenesis of neurodegenerative diseases. Insufficiency of the chaperone function of Sigma1R and BiP depending on the duration and severity of the pathological process is the common link of neurodegenerative diseases’ pathogenesis highlighted in the review. Therefore, the search for neuroprotective agents among Sigma1R chaperone ligands and modulators that promote protein folding activation and adaptive UPR is promising [137,148,357,358,359].

Table S1 characterizes the neuroprotective effects of Sigma1R ligands established in experimental models of neurodegenerative diseases in vitro and in vivo. Several Sigma1R ligands have been included in clinical trials. Donepezil, which has Sigma1R agonist and noncovalent reversible inhibitor of acetylcholinesterase (AChE) properties [360,361], is undergoing phase 3 clinical trials designed to evaluate the drug’s effect on AD symptoms after 6 months of treatment (NCT04661280). Pridopidine, a dopamine stabilizer that is a D_2_ receptor antagonist and agonistically affects Sigma1R [345,362,363], is included in phase 3 clinical trials for patients with early stage HD (NCT04556656) and phase 2/3 for participants with ALS (NCT04615923). The Sigma1R agonist and muscarinic receptor modulator ANAVEX2-73 (blarcamesine) [181,364] is in a phase 2/3 study for patients with early AD (NCT04314934, NCT03790709), phase 2 for PD patients with dementia (NCT04575259). Compound T-817MA [148,175,365] is included in a phase 2 study to evaluate its efficacy and safety in patients with mild cognitive impairment due to AD (NCT04191486).

The anxiolytic afobazole, a Sigma1R ligand with agonist-like actions [269,366,367], introduced into medical practice in Russia, exhibits cytoprotective and neuroprotective properties in a number of experimental models [368,369,370,371,372,373,374,375,376,377,378,379,380] (Table S1).

In addition to Sigma1R ligands, modulators of ligand binding and functional activity of Sigma1R are distinguished [186,381]. Thus, co-coupling of OZP002 exhibits neuroprotective properties in the Aβ_25–35_ mouse model of AD [186].

The pharmacology of Sigma1R has revealed several important features that allow its chaperone activity to be fine-tuned depending on the disease pathogenesis. As noted by T. Maurice, Sigma1R ligands exert bi-phasic (bell-shaped) dose–response effects on pathogenetic processes without affecting physiological functions in vitro and in vivo [169,178,357]. Moreover, Sigma1R ligands have an individual profile of pharmacological action in close dose ranges, which forces us to go beyond the classical notions of Sigma1R agonists and antagonists. For example, NE-100, referred to as a Sigma1R antagonist, in experiments on HT-22 cells reduced ER stress-induced cell death caused by tunicamycin but not by glutamate in a concentration-dependent manner. At the same time, Sigma1R antagonist BD-1047 had no effect on tunicamycin-induced cell death in a wide range of concentrations [172]. Compounds donepezil and (+)-igmesine with Sigma1R agonist activity promoted BDNF secretion from the neuronal MN9D cells, whereas Sigma1R agonist PRE-084 had no such effect [382]. PRE-084 and Sigma1R antagonist BD-1063 reduced spinal motor neurons degeneration in SOD1 p.G93A female mice, whereas agonist SA4503 did not [326]. It is possible that these properties result from the ability of Sigma1R to exhibit different affinity to the ligand depending on the homomeric structure of the chaperone [383,384,385,386], which may vary in the dynamics of neurodegenerative diseases’ pathogenesis. In addition, it is not yet clear whether all compounds classified as agonists induce the conformational changes of Sigma1R similar to those of the prototypical agonist (+)-pentazocine [37].

The above data point to the possibility of selecting the optimal activator of Sigma1R chaperone function to be included in the therapeutic strategy of a particular neurodegenerative disease. It can be assumed that neuroprotective properties of Sigma1R ligands in models of neurodegenerative diseases characterized above are attributed to the chaperone activity of Sigma1R towards target proteins of various cellular compartments, including MAM-resident proteins associated with UPR signaling. The regulatory effects of activated Sigma1R may result in the restoration of structure and function of MAMs and MERCs, enhancement of BDNF-dependent neuroplasticity, and attenuation of neuroinflammation.

### 7.2. Contribution of Sigma1R-Dependent MAM Regulation to Neuroprotective Activity

It has been shown on the example of AD pathogenesis that the accumulation of plaques and tangles in neurons occurs later than disorders of mitochondrial function, calcium homeostasis and lipid metabolism [387,388]. Disorders of MAM structure and functions of resident proteins are currently viewed as the early stages of neurodegenerative diseases pathogenesis [33,116,117,388,389,390,391,392,393].

High levels of presenilins, APP, and γ-secretase, proteins which mutations are responsible for FAD, have been demonstrated in MAM [5,153,155,156], indicating that APP to Aβ processing can occur not only in the plasma membrane but also in MAM [394]. Thus, MAM lipid rafts disturbances are capable of enhancing Aβ production and increasing the ratio of Aβ_42_/Aβ_40_ [387,395,396]. On the other hand, the accumulation of γ-secretase substrate and Aβ precursor (C99) in MAM contributes to the upregulation of MAM-resident functions, impaired cholesterol trafficking and mitochondrial function [391,397,398].

Proteins included in the PD pathogenesis are expressed in MAMs [249,250,251]. These include the α-Syn protein [262,399], which is essential for maintaining mitochondrial Ca^2+^ homeostasis and regulating ER-mitochondria connection [400]. Similar to α-Syn, the DJ-1 protein (PARK7) is expressed in MAM [263,401]. Together with IP_3_R3, GRP75, and VDAC1, it forms a macrocomplex that ensures ER-mitochondria coupling and Ca^2+^ transport [263]. The structure and function of MAMs have been shown to depend on the activity of the *PRKN* [402,403]. PINK1 protein is found in the MAMs of dopamine-positive SH-SY5Y cells [404].

MAM contains a high level of proteins which dysfunction is responsible for ALS pathogenesis [312,313,314]. Misfolded ALS-causing dismutase active p.G93A and dismutase inactive p.H46R hSOD1 mutants have been shown to specifically interact with VDAC1 in spinal cord neurons and reduce its channel conductance [405]. MAM disruption caused by hSOD1 p.G85R (rs121912436) overexpression has been demonstrated in in vitro and in vivo studies [127,406]. Overexpression of WT and mutant protein FUS contributes to the disruption of the VAPB-PTPIP51 ER-mitochondria tethering complex, thus reducing ER-mitochondria associations [407]. Similarly, expression of TDP-43 reduces the binding of VAPB to PTPIP51 and ER-mitochondria associations [408,409].

Reduced levels of GRP75 and IP_3_R3 proteins involved in the formation of MERCs were detected in putamen postmortem samples from HD patients [410]. These data are consistent with a decrease in GRP75 and IP_3_R3 content and the number of MERCs in HD simulations both in vivo and in vitro [344,346,410,411,412]. The contribution of MAM-resident proteins to the pathogenesis of HD is comprehensively covered in the review [413].

It was shown that MERCS and MAM-induced impairment of mitochondrial bioenergetic function can be prevented by ligand activation of the Sigma1R chaperone. As indicated above, the Sigma1R (+)-pentazocine agonist under ER stress prolongs the association of Sigma1Rs with IP_3_R3s in MAM, thus preventing IP_3_R3s degradation, maintaining normal MAM morphology, mitochondrial Ca^2+^ uptake, and ATP production [107,121].

Sigma1R ligands regulate MAM levels in neural progenitor stem cells (hNPCs) constitutively overexpressing human APP_Swe_. The Sigma1R agonist PRE-084 (10 μM) increased total IP_3_R3 levels by 43%, whereas treatment of hNPCs with antagonist NE-100 (10 μM) decreased total IP_3_R3 levels by 20% versus vehicle control cells. At the same concentrations, PRE-084 increased MAM-associated IP_3_R3 levels by more than 3.5-fold, whereas NE-100 more than halved this index. Moreover, PRE-084 stabilized while NE-100 reduced the stability of palmitoylated APP (palAPP) which is preferentially targeted to lipid raft domains where it serves as a good BACE1 substrate [124,414]. Palmitoylation inhibitors severely impair the processing of APP by α- and β-secretases [414].

In an in vitro ALS model, PRE-084 (5μM) restored the function of IP_3_R3 disrupted by mutant SOD1, namely, it reduced Ca^2+^ flux in cytoplasm, increased Ca^2+^ flux in mitochondria, reduced calpain activity, and increased ATP levels. The observed effects suggest that Sigma1R activation by PRE-084 prevented the disruption of the Sigma1R-IP_3_R3 complex. Moreover, PRE-084 in presymptomatic hSOD1 p.G93A mice successfully restored co-localization of Sigma1R and IP_3_R3 in anterior horn neurons of the lumbar spinal cord [127].

The above data indicate the possibility of achieving neuroprotective effects by Sigma1R ligands with agonist properties by activating the chaperone functions of Sigma1R in relation to IP_3_R3 and restoring the morphology of MAMs and MERCs, Ca^2+^ homeostasis and ATP synthesis.

### 7.3. Contribution of Sigma1R-Dependent Regulation of UPR and BDNF Signaling to Neuroprotective Activity

As mentioned above, in 2013, a group of researchers led by T.-P. Su discovered the transient association of Sigma1R with IRE1 at the beginning of ER stress in cells’ MAM, which resulted in the formation of a conformationally stable and long-lasting IRE1 endonuclease and the production of active XBP-1s [108]. Given the previously established ability of Sigma1R agonists to induce dissociation of the Sigma1R-BiP complex [107], this research group hypothesized the contribution of activated Sigma1R to the neuroprotective activity of Sigma1R ligands by sustaining and prolonging the IRE1 signaling pathway [108]. This assumption was confirmed and developed in the studies led by I. Bezprozvanny, who was first to prove the contribution of Sigma1R chaperone interaction with cholesterol to the regulation of IRE1α signaling activity [123].

This assumption is supported by the association of AD and bipolar disorder with G allele of the *XBP1* rs2269577 SNP, which causes lower XBP1-dependent transcriptional activity [415,416]. XBP-1 deficiency in the nervous system causes functional deficits in hippocampal synapses, which lead to learning and memory impairment in mice. On the contrary, enhanced expression of the active XBP-1s form restores LTP in hippocampal slices as well as motor and behavioral performance [417]. Disorders in mice with XBP-1 deficiency in the nervous system were found to be caused by a significant hippocampal-specific decrease in Brain-derived neurotrophic factor gene (*Bdnf*) expression [417,418]. Induction of XBP-1s expression in the hippocampus upregulates *Bdnf*. An inverse activating effect of BDNF on XBP-1 has also been shown [417,419]. Enhancement of *Xbp1* mRNA splicing was eliminated by the specific inhibitor of IREα STF-083010, indicating an important role of IRE1α activation in the physiological processes of neuroplasticity and learning [417].

The above results are in agreement with the research of A. Saito et al. where glutamate-induced IRE1-dependent activation of XBP-1 in the dendrites of primary mouse hippocampal neurons facilitates *Bdnf* transcription in the soma. It was shown that the activating effect of BDNF on IRE1α/XBP-1 pathway is performed in a PKA-dependent manner [420]. The authors of these studies similarly consider the activation of the IRE1α/XBP-1 pathway to be an important element in the positive feedback loop of BDNF-dependent regulation of synaptic plasticity, learning and memory-related processes [417,420].

It cannot be ruled out that these BNDF-dependent processes are triggered by Sigma1R agonists through the activation of Sigma1R chaperone function towards IRE1α. For instance, the enhancement of neurite outgrowth in vitro induced by PRE-084 is prevented by TrkB inhibitor K252a [421]. Two weeks of cutamesine (SA4503) administration to Wistar rats increased BDNF content in the hippocampus [422]. Agonist effect on Sigma1R also increased BDNF level during CNS damage modeling in vivo. The neuroprotective activity of pridopidine in HD modeling on R6/2 mice was accompanied by an elevation of BDNF expression in the striatum [350]. The neuroprotective effects of PRE-084 and pridopidine in the 6-OHDA model of PD were similarly accompanied by an increase in BDNF levels and activation of BDNF-dependent signaling pathways in the striatum [264,265]. In bilateral common carotid artery occlusion (BCCAO) mice, chronic PRE-084 administration eliminated learning and memory deficits and neuronal cell damage in the hippocampus, which was combined with an increase in BDNF levels and TrkB phosphorylation. The effect of PRE-084 was reversed by Sigma1R antagonist BD-1047 [423].

Fluoxetine and imipramine, which possess Sigma1R agonist properties, exhibited neuroprotective effects when PD was simulated by MPTP administration to mice or exposure of SH-SY5Y cells to MPP^+^. The effects of both drugs were combined with their ability to increase the BDNF level and glial cell line-derived neurotrophic factor (GDNF) in the SNc and striatum of mice and activate BDNF signaling in vitro [274]. The neuroprotective activity of imipramine in the rotenone model of PD in rats was also combined with the enhanced striatal *Bdnf* expression [276].

The anxiolytic afobazole increased the BDNF level in the hypothalamus and hippocampus of BALB/c and C57Bl/6 mice 1 h after injection compared to the vehicle. In BALB/c mice afobazole prevented stress-induced decrease in BDNF level in the hypothalamus, hippocampus and striatum [424]. In in vitro experiments afobazole increased BDNF level in HT-22 cell culture [425].

It is important to note the ability of activated chaperone Sigma1R to interact with TrkB [421], inhibit BDNF aggregation [130], stimulate posttranslational processing and BDNF secretion in vitro and in vivo [130,382,426]. These data allow us to consider ligand activation of Sigma1R as an efficient approach to neuroprotective pharmacotherapy by restoring BDNF-dependent brain plasticity.

### 7.4. Contribution of Sigma1R-Dependent Regulation of UPR and Neuroinflammation to Neuroprotective Activity

Under physiological conditions, interleukins play an important role in brain development, ensuring neuroplasticity and related learning and memory processes. However, neuroinflammation caused by the activation of glial cells and increased production of interleukins promotes aging and considered to be a link in the neurodegenerative diseases’ pathogenesis [21,427,428]. In the interleukin family, IL-1β and IL-6 play an important role in the induction of neuroinflammation [429,430].

In vitro experiments aimed to study the role of Sigma1R in IRE1-dependent mechanisms of inflammation using proximity ligation assay confirmed the ability of Sigma1R to interact with IRE1, which was enhanced in the presence of LPS [133]. In *Sigmar1^−/−^* mouse bone marrow-derived macrophages (BMDM) and liver homogenates, a significant enhancement of LPS-induced but not basal XBP-1 splicing was detected [133], indicating that Sigma1R restrains the activation of IRE1/XBP-1 signaling pathway which is consistent with the results of s1r^+25/+25^ mutant zebrafish lines [134]. LPS-induced activation of IRE1/XBP-1 signaling in *Sigmar1^−/−^* BMDM was found to increase the expression of the proinflammatory cytokine IL-6, which was blocked by the IRE1α inhibitor 4μ8C [133]. IL-6 plays a key role in the activation of microglia and astrocytes under neuroinflammation. Its levels get elevated in the brain during aging, as well as in patients with depression, learning and memory disorders [427,431]. On the other hand, overexpression of *SIGMAR1* in HEK 293 cells caused a decrease in the expression of the proinflammatory cytokine IL-8 [133], which production increases during neurodegenerative diseases [432].

Sigma1R ligands with agonist activity were found to reduce proinflammatory cytokine levels in models of neurodegenerative diseases and in clinical studies. SSRIs fluvoxamine and fluoxetine, which possess properties of Sigma1R agonists [267], prevented LPS-induced increase in IL-6 levels in mice serum and increase in IL-1β, TNFα and iNOS in the SN of MPTP-treated mice [133,273]. The effect of fluvoxamine was not evident in *Sigmar1^−/−^* mice. Fluvoxamine similarly reduced LPS-induced levels of IL-6, IL-1β, IL-12 p40, and IL-8 in peripheral blood from healthy donors [133]. It has been shown that the effects of fluvoxamine may be related with both Sigma1R chaperone function activation [267], and its ability to enhance *Sigmar1* expression [433]. Fluoxetine and imipramine prevented MPTP-induced death of dopaminergic neurons by inhibiting microglial activation in the SNc [273,274], which corresponds to Sigma1R agonist PRE-084 action [264]. Imipramine reduced striatal TNFα level and iNOS protein expression in the rotenone model of PD in rats [276].

Chronic unpredictable mild stress caused an increase in IL-6 and TNFα levels in rat cardiomyocytes, which was relieved by chronic cutamesine (SA4503) administration [434]. The prototype Sigma1R agonist (+)-pentazocine reduced the NMDA-induced production of IL-8 and NO by Jurkat cells to control values [435].

Afobazole attenuates the migration of microglia and elevations in [Ca^2+^]_i_ elicited by ATP and UTP in vitro [436]. Afobazole also exhibited protective effects, attenuating the membrane ruffling, chemotaxis, expression of activated caspase-3, and cell death induced by exposure of primary microglia cultures to Aβ_25–35_ [177]. Similar to the Sigma1R agonist effects described above, afobazole had the ability to reduce the secretion of proinflammatory IL-2, IL-4 and INFγ induced phytohaemagglutinin in human T-lymphocyte culture [437].

It was shown that effects of Sigma1R ligands with agonist properties are consistent with the protective effects on cells associated with the reduction of IRE1α activity. The inhibitory effect of 4μ8C [133] on IL-6 expression is similar to the ability of another IRE1α inhibitor, STF-083010, to prevent the development of atherosclerosis by reducing proinflammatory protein expression [438]. STF-083010 also reduced Aβ_1–40_-induced caspase-2 activation and SH-SY5Y cell death [439]. The neuroprotective properties of Sigma1R ligands and IRE1α inhibitors presented in a number of studies are consistent with the effects of attenuating gene expression of proteins providing IRE1α signaling in models of AD [213], PD [440,441], ALS [98,442,443], and HT [103].

Thus, current data indicate the ability of Sigma1R to restrain IRE1α activity when inflammation occurs. This property correlates with the ability of Sigma1R agonists to inhibit neuroinflammation and exhibit neuroprotection in experimental models of neurodegenerative diseases.

### 7.5. Possible Role of BiP in Sigma1R-Dependent Neuroprotection

As noted above, ligand activation of Sigma1R promotes the dissociation of the Sigma1R-BiP complex and activation of both chaperones [107]. The hypothesis, which was proposed 20 years ago in the work of T. Hayashi et al., regarding the important role of BiP induction in ER stress weakening and enhancing cell survival [444], is now sufficiently confirmed in the context of studying the neuroprotective mechanisms in the models of neurodegenerative diseases. M. Kopp et al. [64] with reference to proteomic studies [445,446] noted the prevalence of BiP expression over other proteins in the ER, which makes it difficult to regulate BiP activity through interaction with its client proteins. Taking into account the fact that Sigma1R is predominantly expressed in the MAM [108], one would expect a greater effect of Sigma1R-BiP complex dissociation on the activation of the latter in this cellular compartment. On the other hand, it is possible that similarly to the reduction of aggregation and degradation of IP_3_R3 in complex with Sigma1R [107], interaction of Sigma1R with BiP can prevent degradation of relatively short-lived BiP [278]. The above arguments are consistent with the opinion of S. Preissler et al. on the role of BiP oligomerization in providing a cell with a BiP reserve, which can be used in enhancing cellular stress without activating the translational and transcriptional mechanisms of the UPR [447]. Thus, ligand activation of Sigma1R can contribute to a rapid increase in free BiP levels and the folding capacity of MAM proteins associated with neurodegenerative diseases.

In addition, the dependence of BiP expression on IRE1α-XBP-1 signaling is known [70]. For example, in cells expressing IRE1 derivative (∆IRE) with truncated cytoplasmic region and thus lacking the kinase and RNase L domains, decreased *HSPA5* (*BiP*) mRNA level and increased vulnerability to ER stress stimulated by tunicamycin are shown. This effect is reversed by infection with recombinant BiP [94]. This result is consistent with the neuroprotective effect of recombinant human Hsp70 in the bilateral olfactory bulbectomy and 5XFAD mouse models of AD [448]. Therefore, it is possible that BiP levels increase is related to the stabilizing effect of Sigma1R on IRE1α and XBP-1-dependent chaperones expression [70,108,133].

These examples of possible Sigma1R-dependent mechanisms of UPR regulation correspond to both the above characterized neuroprotective effects of Sigma1R ligands with agonist activity and the neuroprotective activity that arises when BiP expression is increased. It was shown that the onset of neurodegenerative diseases is closely associated with aging, which is accompanied by a decrease in proteostasis network capacity [44], including a decrease in *Hspa5* (*BiP*) expression [449,450,451]. On the other hand, spatial learning of C57Bl/6J mice induced the expression of *Hspa5* in the hippocampus as soon as within one hour [452].

Studies of neurodegenerative diseases’ pathogenesis have revealed the association of BiP with the protection of neurons under disrupted protein folding conditions. Thus, BiP contributes to the decrease in APP, Aβ_40_, and Aβ_42_ secretion during ER stress modeling through its interaction with APP and reduction of APP availability to β- and γ-secretases [237,238]. In addition, BiP inhibits Aβ fibrillation by maintaining its structure in the monomer state [453], stimulates uptake of Aβ_1–42_ in microglia [454] and exerts anti-excitotoxic and anti-apoptotic actions in neurons via suppression of oxidative stress and stabilization of calcium homeostasis [455]. Increased expression of BiP protects SH-SY5Y cells from apoptosis induced by WT α-Syn overexpression [285]. In experiments on rats BiP overexpression prevented the loss of TH+ neuronal cells in the SNc, maintained striatal DA level and eliminated behavioral deficit in the α-Syn PD model [280,285]. In a rotenone model of neurotoxicity in vitro, application of miR-384-5p inhibitor caused BiP expression enhancement, which was combined with attenuation of ER stress sensors activation and decreased death of SH-SY5Y cells and primary human neurons [456]. A recent study demonstrated the ability of BiP to bind to the TDP-43 protein, which accumulates in motoneurons of the spinal cord of ALS patients. Overexpression of the Drosophila BiP homologue Hsc70.3 in the Drosophila eye mitigated TDP-43-induced toxicity [457]. Upregulation of BiP in the Neuro2a cells efficiently prevented aggregate formation, reduced caspase-12 levels, caspase-3 activity, and increased Htt150Q cell viability. Silencing of BiP expression by siRNA caused an inverse effect on these parameters [354]. Ectopic expression of BiP, which binds to luminal region of IRE1 and thus blocks oligomerization and activation of IRE1, reduced IRE1-mediated Htt aggregation in Htt120Q SH-SY5Y cells [103].

BiP expression activators 2-deoxy-D-glucose (2-DG) and BiP inducer X (BIX) have cytoprotective and neuroprotective properties in vitro and in vivo [458,459,460,461]. It was shown that enhancement of BiP expression mediates the protective effect on neurons by activation of folding and prevention of prolonged or excessive activation of ER stress sensors contributing to cell apoptosis [444], which is consistent with a short-term stimulatory effect of BiX on BiP expression with a maximum after 4 h, in contrast to long-term activation of chaperone expression by tunicamycin [459]. The revealed protective effect of Hsp70 expression inducer in the MPTP model of PD in mice [462] confirms the feasibility of BiP activation for neuroprotective activity achievement.

## 8. Conclusions

This review analyzes the contribution of chronic ER stress caused by deficiencies in chaperone function of Sigma1R and BiP to the pathogenesis of neurodegenerative diseases. As shown by the literature analysis, changes in the expression of Sigma1R and BiP chaperones detected both in clinical studies and in experimental modelling of neurodegenerative diseases have common and specific features. Thus, patients with AD, PD, and ALS are characterized by a decreased expression of Sigma1R in the brain, while BiP chaperone content changes multidirectionally. Such data may indicate a weakened chaperone function of Sigma1R in the pathogenesis of these diseases. On the other hand, no Sigma1R downregulation was detected in the brain of HD patients and in HD simulations in vivo against the background of increasing BiP levels, which is considered to be a marker of ER stress. However, these results are not reproduced in most transgenic HD models in vitro. In contrast, no increase in BiP expression was detected after transfection of genetic constructs with causative AD mutations into cell cultures, which can be considered as an absence of ER stress induction. Nevertheless, similar patterns are not observed in transgenic models of PD. It is plausible that these results indicate a different contribution of AD or PD causative genes expression to ER stress. In in vivo and in vitro models of ALS, BiP content predominantly increases in in vitro experiments and changes insignificantly in in vivo models along with the absence of Sigma1R expression amplification (Figure 1, Figure 2, Figure 3 and Figure 4). It is possible that in a number of cases the above-mentioned correlations reflect the reproduction of different stages of the pathogenesis of neurodegenerative diseases. In addition, chaperone expression peculiarities should be taken into account when considering the translationality of transgenic experimental models.

In contrast to transgenic models of AD in vitro, AD and PD modeling by Aβ peptides or toxins, respectively, in most experiments is accompanied by an increase in BiP content while no induction or attenuation of Sigma1R expression is recorded (Figure 1, Figure 2, Figure 3 and Figure 4). It is possible that the identified features of chaperone expression under simulation of specific neuronal damage by toxic agents or mutant protein production conditions will contribute to a better understanding of the differences in the pathogenesis of genetically determined and sporadic forms of neurodegenerative diseases.

**Figure 5:**
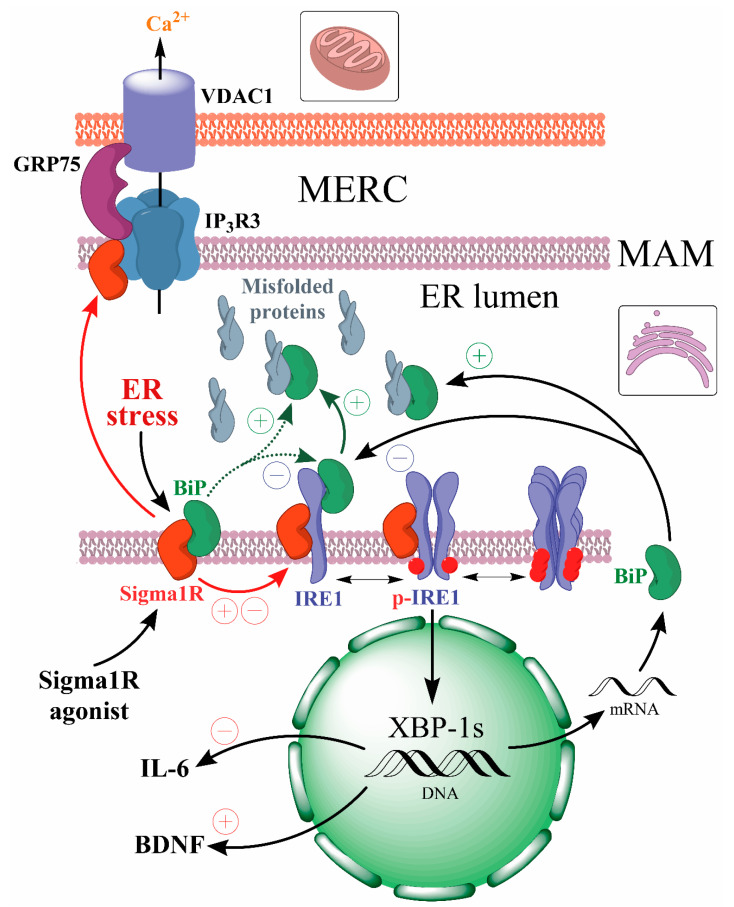
Possible mechanisms of Sigma1R-dependent neuroprotection through UPR regulation. Sigma1R activation under ER stress conditions or Sigma1R ligands with agonist properties action causes dissociation of the Sigma1R-BiP chaperone complex. The dissociation of the Sigma1R-BiP complex enhances the chaperone activity of BiP on misfolded proteins and the regulatory effects of Sigma1R on client proteins (IP_3_R3, IRE1α). The regulatory effects of activated Sigma1R on IRE1α cause an increase in BiP and BDNF expression and a decrease in proinflammatory interleukin expression. Thus, Sigma1R activation contributes to endogenous neuroprotective mechanisms aimed at attenuating ER stress and neuroinflammation while enhancing neuroplasticity. 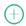
—BiP activation, 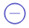
—IRE1α inhibition, 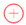
—Sigma1R-dependent positive regulation, 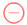
—Sigma1R-dependent negative regulation, 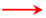
—Translocation of activated Sigma1R, 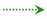
—hypothetical fast Sigma1R-dependent BiP activation.

It was shown that the activation of Sigma1R and BiP chaperones, which promotes increase in the protein folding capacity and adaptive UPR can be considered as an approach to pharmacotherapy of neurodegenerative diseases associated with accumulation of aberrant proteins (Figure 5). In this review, we attempt to justify an assumption that neuroprotective effects of Sigma1R ligands are achieved by activation of Sigma1R and BiP chaperones through a dissociation of Sigma1R-BiP complex in ER-mitochondria contact region in the concert with multidirectional regulation of IRE1α-dependent UPR signaling. The validity of this assumption is confirmed by the similarity of protective cellular mechanisms induced by both ligand activation of Sigma1R and upregulation of BiP-dependent signaling, that can be observed, for example, following the analysis of cytoprotective effects of afobazole [177,269,270,271,367,380,424,436,463,464,465,466,467].

Relationship between Sigma1R and BiP activation is a subject for further investigations. However, the data presented in this review suggest the agonist regulation of Sigma1R as a viable strategy for neuroprotective pharmacotherapy.

## Data Availability

Not applicable.

## Supplementary Materials

The supporting information can be downloaded at https://www.mdpi.com/article/10.3390/ijms24010823/s1. References [127,135,157,168,173,174,175,176,177,178,179,180,181,182,183,184,185,186,187,188,193,194,195,256,264,265,268,270,271,272,273,274,276,324,326,327,346,347,348,350,351,468,469,470,471,472,473] are cited in the supplementary materials.

## Abbreviations

6-OHDA	6-hydroxydopamine; 5-(2-Aminoethyl)benzene-1,2,4-triol
Aβ	Amyloid-β
AD	Alzheimer’s disease
ALS	Amyotrophic lateral sclerosis
ATF4	Cyclic AMP-dependent transcription factor ATF-4
ATF6	Cyclic AMP-dependent transcription factor ATF-6 alpha
BDNF	Brain-derived neurotrophic factor
*Bdnf*	Rodent BDNF gene
BiP	Endoplasmic reticulum chaperone BiP; GRP78
CHOP	DNA damage-inducible transcript 3 protein
D2 receptor	dopamine receptor D2
eIF2α	Eukaryotic translation initiation factor 2 subunit-α
ER	Endoplasmic reticulum
ERAD	Endoplasmic reticulum-associated protein degradation
FAD	Familiar Alzheimer’s disease
GRP75	Stress-70 protein, mitochondrial
HD	Huntington’s disease
*HSPA5*	Human heat shock protein family A (Hsp70) member 5 gene; human chaperone BiP gene
*Hspa5*	Rodent heat shock protein family A (Hsp70) member 5 gene; rodent chaperone BiP gene
hSOD1	Human Superoxide dismutase 1
iNOS	Nitric oxide synthase, inducible
IP3R3	Inositol 1,4,5-trisphosphate receptor type 3
IRE1	Serine/threonine-protein kinase/endoribonuclease IRE1
LPS	Lipopolysaccharide
LTP	Long-term potentiation
MAM	Mitochondria-associated membrane
MERC	mitochondria-endoplasmic reticulum contact
miRNA	Small non-coding microRNA
MPP^+^	1-Methyl-4-phenylpyridin-1-ium
MPTP	1-Methyl-4-phenyl-1,2,3,6-tetrahydropyridine
nNOS	Nitric oxide synthase, brain
NMDA	(2R)-2-(methylamino)butanedioic acid
NO	Nitric oxide
PBMCs	Peripheral blood mononuclear cells
PD	Parkinson’s disease
PERK	Eukaryotic translation initiation factor 2-alpha kinase 3
PET	Positron emission tomography
SAD	Sporadic Alzheimer’s disease
Sigma1R	Sigma nonopioid intracellular receptor 1; chaperon Sigma1R
*SIGMAR1*	Human Sigma1R gene
*Sigmar1*	Rodent Sigma1R gene
*sigmar1*	Zebrafish Sigma1R gene
SN	Substantia nigra
SNc	Substantia nigra pars compacta
SOD1	Superoxide dismutase 1; Superoxide dismutase [Cu-Zn]
SSRI	Selective serotonin reuptake inhibitor
TDP-43	TAR DNA-binding protein 43
trkB	BDNF/NT-3 growth factors receptor; neurotrophic receptor tyrosine kinase 2
UPR	Unfolded protein response
VAPB	Vesicle-associated membrane protein-associated protein B/C
VDAC1	Voltage-dependent anion-selective channel protein 1
XBP1	X-box-binding protein 1
