# Supplementary material for: Chaperone-Dependent Mechanisms as a Pharmacological Target for Neuroprotection
Source: Int J Mol Sci. 2023 Jan 3;24(1):823. doi: 10.3390/ijms24010823 (PMC9820882; doi:10.3390/ijms24010823)
Supplement: Supplementary file 1 [file ijms-24-00823-s001.zip › ijms-2106466-supplementary.pdf]

Table S1. Neuroprotective effects of Sigma1R ligands in in vitro and in vivo models of neurodegenerative diseases.

Compound	Description	Pathology	Experimental model	Effects of the compound	Reference
Donepezil	Sigma1R agonist, acetylcholinesterase inhibitor	AD	Mice injected i.c.v. with $A\beta_{25-35}$ peptide	Anti-amnesic and neuroprotective effects due to blocking of lipid peroxidation in the hippocampus. Doses and route of administration: 0.5 and 1 mg/kg i.p. 20 min before the test on day 7-8 after $A\beta_{25-35}$.	[1]
				Prevented the $A\beta_{25-35}$-induced spontaneous alternation deficits or passive avoidance deficits. Doses and route of administration: injected at 0.5 mg/kg i.p.20 min before the peptide (9 nmol ICV), 7 days before the measure of spontaneous alternation and 8 days before the passive avoidance training, retention being tested after 24 h.	[2]
			Mouse forebrain mitochondria incubated with $A\beta_{1-42}$ peptide	Attenuates $A\beta_{1-42}$-induced alteration in ROS production (1 and 3 μM).	[3]
			Rat cortical neuronal cells incubated with $A\beta_{42}$	Recovers PP2A activity in a concentration dependent manner (0.1, 1, 10, and 100 μM for 24h, followed by 20μM $A\beta_{42}$ for 6h). Regulates PP2A methylation/demethylation (pre-treatment with 10μM). Affects the level of tau phosphorylation at S-396 sites (pre-treatment with 10μM).	[4]
				Antiamnestic and neuroprotective effects. Doses and route of administration: 1 mg/kg i.p. 20 min before the test on day 7-8. Effects of PRE-084 were blocked with BD-1047 at a dose of 0.5–1 mg/kg i.p. 20 min before the test on day 7-8.	[1]
				Prevented the $A\beta_{25-35}$-induced spontaneous alternation deficits or passive avoidance deficits. Doses and route of administration: injected at 0.3 mg/kg i.p.20 min before the peptide (9 nmol ICV), 7 days before the measure of spontaneous alternation and 8 days before the passive avoidance training, retention being tested after 24 h.	[2]
				Increases mitochondrial respiration rates. Prevents $A\beta_{25-35}$-induced increases in lipid peroxidation levels. Decreases Bax/Bcl-2 ratio and cytochrome C release into the cytosol, thus preventing the mitochondrial respiratory dysfunction and resulting oxidative stress and apoptosis.	[5]

PRE-084	Sigma1R agonist	AD	Mice injected i.c.v. with Aβ_{25-35} peptide	Doses and route of administration: 0.5 mg/kg i.p. 20 min before Aβ_{25-35}, 7 days before sacrifice. The effects were blocked by BD-1047 10 mg/kg i.p. administered simultaneously with PRE-084.	
				Blocks Aβ_{25-35}-induced recognition memory deficits. Prevents alterations in kinase activity (Akt, GSK-3b) and Tau hyperphosphorylation. Attenuates the increase in Aβ_{1-42} content. Doses and route of administration: 0.5 and 1 mg/kg i.p. 20 min before the Aβ_{25-35} or sc.Aβ i.c.v. injection.	[6]
				Protects pyramidal cells of hippocampal CA1 region. Doses and route of administration: 1 mg/kg i.p. once daily on 1-7 days after Aβ_{25-35} injection.	[7]
				Enhances locomotion. Doses and route of administration: 0,3, 1, 3 mg/kg i.p. 30 min before the training and 7 days after i.c.v. inj. with Aβ_{25-35} peptide. Enhances learning. Doses and route of administration: 1 mg/kg. i.p., 30 min before the training and 14 days after i.c.v. inj with Aβ_{25-35} peptide. Effects were blocked by haloperidol at 0,03 and 0,1 mg/kg and BMY-14802 at 10 mg/kg i.p.	[8]
			Mice injected i.c.v. with Aβ_{1-42} peptide	Stimulates hippocampal cell proliferation and differentiation. Reduces Aβ_{1-42}-induced astrogliosis. Doses and route of administration: 1 mg/kg i.p. daily between postsurgery days 7-12.	[9]
			Mouse forebrain mitochondria incubated with Aβ_{1-42} peptide	Weakens Aβ_{1-42}-induced increase in ROS at concentration higher than 1 μM, by preventing decrease in complex I (10 μM) and IV (1 and 10 μM). NE-100 failed to affect ROS levels and complex I and IV activity. Prevents the Aβ_{1-42}-induced decrease of respiration control ratio at 1 μM. NE-100 and progesterone failed to affect respiration control ratio or even worsened it with NE-100 at 10 μM.	[3]
			Rat cortical neurons incubated with Aβ_{25-35} peptide	Reduces neuronal death (10 μM 24 h). Decreases levels of Bax in cortical neurons (10 μM 24 h), NE-100 (5 μM) reverses this effect.	[10]
		PD	MPTP mouse model	Reduces loss of DA neurons. Restores motor ability. Doses and route of administration: 1mg/kg i.p. 3 days in advance, and MPTP was administered from the fourth day. Restores MPTP-induced damage to mitophagy activity in the SNc in a concentration-dependent manner Doses and route of administration: 0.1 μM, 1 μM or 10 μM for 1 h.	[11]
				Restores motor activity. Increases density of dopaminergic fibrils in the most denervated striatal regions. Increases DA levels, upregulates	[12]

	6-OHDA mouse model	neurotrophic factors (BDNF and GDNF) and their downstream effector pathways (extracellular signal regulated kinases 1/2 and Akt). Reduces microglial activation in substantia nigra and striatum. Doses and route of administration: 0.3 or 1 mg/kg injected s.c for 7 or 35 days, starting on the same day as 6-OHDA lesion.	
		Normalizes motor function. Prevents decrease of DA in the striatum. Doses and route of administration: 1.0 mg/kg i.p. daily for 14 days starting 30 minutes after the surgery. Pre-administration of BD-1047 at 3.0 mg/kg 30 min prior agonist abolished the action of PRE-084.	[13]
		Facilitates motor behavior. Restores the intrastriatal DA content. Doses and route of administration: 1.0 mg/kg daily i.p. for 14 days starting 30 minutes after the surgery. Pre-administration of BD-1047 at 3.0 mg/kg 30 min prior agonist abolished the action of PRE-084.	[14]
ALS	Transgenic hSOD1 p.G93A mice	Restores MAM disfunctions, preventing the disruption of the Sigma1R–IP_3R3 interaction. Doses and route of administration: 0.25 mg/kg i.p., 3 times per week from postnatal day 35 to 95.	[15]
		Reduces MN death in vitro in a dose-dependent manner (3 and 30 μM). The antagonist BD-1063 (3 and 30 μM) also prevents MN death, with a highest effect at 3 μM. Preserves neuromuscular function, promotes MN survival and reduces microglial reactivity (0.25 mg/kg i.p.).	[16]
		Improves spinal motoneuron function. Improves locomotor performance. Induces protein kinase C specific phosphorylation of the NR1 subunit of NMDA receptor. Reduces microglial immunoreactivity. Prolongs survival of mice. Doses and route of administration: 0.25 mg/kg i.p. daily during 8 or 4 weeks. Coadministration of BD-1036 at 0.25 mg/kg i.p. daily during 8 weeks reverted the improvement.	[17]
	NSC-34 cells expressing p.P56S VAPB	Reduces mutant VAPB aggregation in a dose-dependent manner and facilitates the degradation of soluble mutant VAPB without affecting the normal level of wild-type proteins (24 h incubation with 20 and 50 μM). Restores mitochondrial activity (24 h incubation with 20 μM).	[18]
HD	PC6.3 neuronal cells overexpressed of WT and mutant huntingtin proteins	Induces expression of Sigma1R protein reduced by mutant huntingtin proteins. Increases cell survival by counteracting caspase-3 and caspase-12 activation. Increases the level of cellular antioxidants by activating the NF-kB pathway and reduces ROS.	[19]

				Increases the calpastatin levels, involved in the regulation of oxidative stress (0.3 μM PRE084 was added 4 h after transfection).	
SA4503	Sigma1R agonist	AD	Mice injected i.c.v. with $A\beta_{25-35}$ peptide	Enhances locomotion. Doses and route of administration: 0,3 mg/kg i.p. 30 min before the training and 7 days after i.c.v. inj. with $A\beta_{25-35}$ peptide. Enhances learning. Doses and route of administration: 0,3 mg/kg i.p. 30 min before the training and 14 days after i.c.v. inj with $A\beta_{25-35}$ peptide. Effects were blocked by haloperidol at 0,03 and 0,1 mg/kg and BMY-14802 at 10 mg/kg i.p..	[8]
		ALS	Transgenic hSOD1 p.G93A mice	Improves motor function and preserves neuromuscular junctions. (0.25 and 1 mg/kg i.p.). Prevents motoneurons death (3 and 30 μM). Reduce microglial reactivity (0.25 mg/kg i.p.).	[16]
AF710B (aka ANAVEX 3-71)	Sigma1R agonist, M_1 agonist	AD	Transgenic rats express the human APP gene carrying both the Swedish and Indiana mutations	Reverses cognitive deficits. Reduces AD-like amyloid pathology by decreasing amyloid plaques, insoluble and soluble cortical $A\beta_{42}$ levels. Possesses anti-inflammatory effect reducing cortical IL-10 mRNA expression levels. Displays synaptogenic activity by restoring both mRNA expression and protein levels of synaptophysin. Doses and route of administration: chronic treatment with 10 μg/kg orally daily for 4.5 months was initiated in postplaque 13-month-old Tg rats and interrupted 5 weeks before behavioral testing.	[20]
			3xTg-AD mice	Mitigates cognitive impairments. Decreases BACE1, GSK3β activity, p25/CDK5, neuroinflammation, soluble and insoluble $A\beta_{40}$, $A\beta_{42}$ plaques and neurofibrillary tangles. Doses and route of administration: 10 μg/kg/day i.p. for 2 months, from 10 to 12 months of age.	[21]
ANAVEX1-41, ANAVEX3-71	Sigma1R agonist, muscarinic acetylcholine receptors ligand	AD	Mouse forebrain mitochondria incubated with $A\beta_{1-42}$ peptide	Weakens $A\beta_{1-42}$-induced increase in ROS (ANAVEX1-41 at 1 and 3 μM, ANAVEX3-71 at 0,3 μM) by attenuating decrease in complex IV activity (ANAVEX1-41 and ANAVEX3-71 at 1 and 10 μM).	[3]
ANAVEX2-73	Sigma1R agonist, muscarinic acetylcholine receptors ligand	AD	Mice injected i.c.v. with $A\beta_{25-35}$ peptide	Blocks recognition memory deficits. Blocks the $A\beta_{25-35}$-induced increase in $A\beta_{1-42}$ and C99 levels in the hippocampus. Blocks Tau phosphorylation. Doses and route of administration: 0,3 and 1 mg/kg i.p.	[6]
				Restores normal respiration rates in mitochondria. Prevents $A\beta_{25-35}$-induced increases in lipid peroxidation levels, (the effect was blocked by BD1047 at 10 mg/kg), Bax/Bcl-2 ratio and cytochrome c release into the cytosol, thus preventing the mitochondrial respiratory dysfunction and	[5]

				resulting oxidative stress and apoptosis. Doses and route of administration: 0.3 and 1 mg/kg i.p. injected 20 min before A$\beta_{25–35}$.	
				Prevented the Aβ25-35-induced spontaneous alternation deficit. Doses and route of administration: injected at 0.3 mg/kg 20 min before the peptide (9 nmol ICV), 7 days before the measure of spontaneous alternation and 8 days before passive avoidance training, retention being tested after 24 h	[2]
Pridopidine	Sigma1R agonist, D2 receptors antagonist, serotonin 5-HT receptors and α2-adrenoceptors ligand	AD	Aβ_{1-42} toxicity in vitro (48 h)	Prevents loss of mushroom spines from Aβ_{1-42} oligomer toxicity in mice hippocampal cultures (100 nM 16 h). Reverses Aβ_{1-42}-induced impairment of LTP in acute mice hippocampal slices (30 nM).	[22]
			Transgenic PS1-KI (p.Met146Val) mice hippocampal neurons	Rescued mushroom spines in PS1-KI cultures (100 nM 16 h). NE-100 and BD-1047 blocked the rescue of mushroom spines.	[22]
		PD	6-OHDA mouse model	Recovers motor activity. Increases striatal DA fibers density. Upregulates neurotrophic factors (GDNF and BDNF), activates ERK1/2 in the striatum. Doses and route of administration: 6-OHDA lesions followed by a 35-day treatment with 0.3 mg/kg pridopidine s.c.	[23]
			6-OHDA rat model	Decreases L-DOPA-induced sensitized locomotor activity. Doses and route of administration: 12 days after 6-OHDA inj. apomorphine test was produced, on the following day inj. of L-DOPA+25 μmol/kg pridopidine twice a day s.c. was started for either 7, 14, 21 days.	[24]
		ALS	Transgenic hSOD1 p.G93A mice	Improves motor activity in the pole test. Doses and route of administration: pridopidine was released through the osmotic pump for 4 weeks, 3.0 mg/kg/day.	[25]
		HD	R6/2 transgenic mice expressing exon 1 of human Htt with approximately 160 ±10 (CAG) repeats	Improves motor activity in pre-symptomatic and symptomatic animals. Reduces the size of single mutant huntingtin (mHtt) aggregates in the striatal tissues at the end of the treatment. Doses and route of administration: 5 mg/kg i.p. was administered to pre-symptomatic mice starting at week 5 to week 11 and for symptomatic animals starting from week 7 to week 9 and 1 week of daily administration (6 mg/kg) at week 10.	[26]
			Immortalized mouse striatal knock-in cells expressing endogenous levels of wild-type	Shows neuroprotective effects by reducing apoptosis and enhancing phosphorylation state of prosurvival kinase ERK (15μM).	[26]

			(STHdh7/7) or mHtt (STHdh111/111)		
			HEK 293 cells were transfected for the expression of myc-Htt96Q STHdhQ7/7 cells are transfected with either Htt96Q or Htt20Q constructs	Reduces mHtt-induced ER stress (150 μM starting at 4 h till 24 post transfection). Increases the sequestration of mutant huntingtin (mHtt) into insoluble aggregates.	[27]
			Primary neurons from YAC128 HD mice expressing the mutant human HTT gene	Induces mitochondrial functions by preventing the disruption of ER-mitochondria contact sites and improving co-localization of IP_3R and its chaperone Sigma1R with mitochondria (incubation for 24 h at 1 μM). Improves mitochondrial anterograde transport (at 1 μM). Reduces mitochondrial ROS levels by normalizing mitochondrial complex activity (pretreated at 5 μM before H_2O_2). Improves motor coordination (30 mg/kg/day orally 45 days).	[28]
			Lymphoblasts derived from HD patients incubated with 0.1 mM H_2O_2	Restores mitochondrial membrane potential (administered 24 h before H_2O_2 at 1 μM)	
T-817 MA	Sigma1R agonist	AD	Rats injected i.c.v. with $A\beta_{1-40}$ peptide	Ameliorates learning deficits. Prevents granule cell loss in the dentate gyrus of the hippocampus. Doses and route of administration: 8.4 mg/kg/day, p.o. 1 h before the training/testing time from the first day of Aβ infusion until the end of the experiment, during 1-5 weeks.	[29]
			Rats injected i.c.v. with $A\beta_{1-40}$ peptide	Ameliorates learning deficits. Induces hippocampal neurogenesis. Doses and route of administration: treatment was started 4 weeks after Aβ infusion at 8.4 mg/kg/day, p.o during 5 or 6 weeks.	[30]
			Coculture of rat cortical neurons with glia incubated with $A\beta_{1-42}$ peptide	Attenuates neurotoxicity (0.1 and 1 μM 48 h).	[31]
		Tau pathology (AD, PD)	Transgenic mice expressing mutation p.P301L of human tau (P301L mice)	Inhibits neuronal degeneration and improves motor and cognitive impairments by preventing the progression of motor deficit, loss of spinal cord motor neurons and attenuating the spatial memory impairment and the reduction in synaptic terminal density in the hippocampal dentate	[32]

				gyrus. T-817 MA was dissolved in distilled water at concentrations of 0.21 and 0.7 mg/mL, and administrated ad libitum drinking.	
Fluvoxamine	Sigma1R agonist, selective serotonin reuptake inhibitor	AD	SK-N-MC neuronal cells	Decreases y-secretase activity of the APP (46 nM NE-100 at 25 nM abrogated this effect).	[33]
			CHO-AβPP cells	Regulates APP processing in CHO-APP cells: decreases Aβ levels (at 10 μM) due to inhibition of y-secretase activity and total protein level (at 100 μM).	
			J20 amyloidogenic mouse model	Improves memory function. Doses and route of administration: 8 months at dose of 10 mg/kg/day.	
		PD	Maternally separated + 6-OHDA rat model	Improves motor function. Decreases basal plasma corticosterone and lipid peroxidation levels. Elevates DA concentration in the prefrontal cortex. Doses and route of administration: 25 mg/kg/day i.p. from PND 29-59.	[34]
Fluoxetine	Sigma1R agonist, selective serotonin reuptake inhibitor	AD	Mice injected i.c.v. with amyloid oligomers	Prevents the over-expression of inducible nitric oxide synthase (iNOS) and NADPH oxidase 2 (Nox2). Restores the mRNA expression of glutathione peroxidase 1 (Gpx1) antioxidant enzyme. Doses and route of administration: 10 mg/kg/day i.p. for a total of 21 to 26 days starting from 7 days before Aβ i.c.v injection.	[35]
			Mixed cultures of cortical cells treated with Aβ oligomers (2 μM) for 48 h	Neuroprotective effect at a concentration of 1μM.	
			AD-like pathology induced under chronic constant light regime in rats	Improves motor activity. Protects against oxidative stress damage. Upregulates expression of molecular markers of circadian rhythm. Rescues $A\beta_{1-42}$ upregulation and AD-related genes dysregulation. Doses and route of administration: 5 mg/kg/day for 4 months.	[36]
		PD		Prevents degeneration of nigrostriatal DA neurons and increases striatal DA levels. Partially recovers motor activity. Protects against oxidative damage by inhibition of expression of proinflammatory cytokines and iNOS, and attenuating microglial NADPH oxidase activation, reactive oxygen species/reactive nitrogen species production. Doses and route of administration: the fluoxetine treatment was done through injection of	[37]

		MPTP mouse model	various doses 2.5 mg/kg/single/day, 5 mg/kg/single/day, 5 mg/kg/twice/day, 10 mg/kg/single/day into the peritoneum at the indicated time points, the first injection was 12 h after the last MPTP injection.	
			Reduces loss of dopaminergic neurons. Ameliorates motor impairment. Prevents DA depletion in the striatum. Reduces dopaminergic neurodegeneration and microglial activation in the nigrostriatal pathway. Upregulates neurotrophic factors. Ameliorates motor deficit. Doses and route of administration: 20 mg/kg/day i.p. during 3 weeks, then MPTP 20 mg/kg × 3 times was injected. Fluoxetine was further injected for 1 week after the MPTP injection.	[38]
		6-OHDA rat model	Improves cognitive deficits by tuning oscillatory activities in the hippocampus. Doses and route of administration: treatment was started 4 and 7 weeks after 6-OHDA inj. at a dose 30 mg/kg, i.p. 2 h before behavioral tests.	[39]
	HD	R6/1 transgenic mice expressing exon 1 of human Htt with approximately 115 (CAG) repeats	Improves cognitive function. Reduces deficits of neurogenesis, volume loss in the dentate gyrus. Reverses a depressive phenotype. Doses and route of administration: 10 weeks treatment with 20 mg/kg/day i.p.	[40]
Imipramine	AD	Mice injected i.c.v. with $A\beta_{25-35}$ peptide	Prevents memory deficits. Demonstrates anti-inflammatory and neuroprotective effects. Inhibits the TNF-increase in the frontal cortex. Decreases the elevated levels of Aβ both in frontal cortex and hippocampus by preventing the decrease of CTFs in the cortex and increase of APP in the hippocampus. Doses and route of administration: treatment was started a day after Aβ 25-35 injection at a dose of 10 mg/kg i.p., the effects were obtained after 9 and 10-14 days of treatment.	[41]
	PD	MPTP mouse model	Reduces dopaminergic neurodegeneration by suppressing the loss of dopaminergic neurons in the substantia nigra and preventing the depletion of DA in the striatum. Reduces microglial activation in the nigrostriatal pathway. Ameliorates motor deficit. Doses and route of administration: 20 mg/kg/day i.p. during 3 weeks, then MPTP 20 mg/kg × 3 times. Imipramine was further injected for 1 week after the i.p. MPTP injection.	[38]

	Sigma1R agonist, selective serotonin reuptake inhibitor		Rats injected s.c. with rotenone.	Restores motor function. Prevents neuronal degeneration: Restores striatal monoamines and brain derived neurotrophic factor levels; Reduces oxidative damage, microglial activation as well as expression of proinflammatory cytokines and inducible nitric oxide synthase. Doses and route of administration: 10 mg/kg, i.p. daily for 35 consecutive days starting 2 weeks prior to rotenone administration. On the 15th day from the start of the experiment, rats received rotenone 1.5 mg/kg 1 hour after imipramine every other day for 3 weeks for a total of 11 injections.	[42]
		HD	Rats injected i.p. with 3-nitropropionic acid (3-NP)	Improves motor function. Attenuates lipid peroxidation, nitrite concentration. Restores SOD and catalase enzyme activities. Restores mitochondrial complex enzyme II and mitochondrial redox activity. Doses and route of administration: 20 mg/kg p.o. followed by 3-NP 10 mg/kg, i.p. for 14 days.	[43]
OZP002	Sigma1R positive modulator	AD	Mice injected i.c.v. with $A\beta_{25-35}$ peptide	Prevents scopolamine-induced learning deficits. Decreases ROS levels, lipid peroxidation, Bax, TNFα and IL-6 levels. Prevents reactive astrogliosis and microgliosis in the hippocampus. Upregulates synaptophysin level and choline acetyltransferase activity. Doses and route of administration: injected i.p. once 20 min before the $A\beta_{25-35}$ peptide (9 nmol i.c.v.), 8 days after animals were produced to tests and then sacrificed for biochemical analyses.	[44]
			Tg2576 mice overexpressing APP_{Swe}	Prevents learning deficits. Decreases lipid peroxiidation, Bax and TNFα levels. Doses and route of administration: 3 times a week 2 mg/kg i.p. during 2 months.	
Afobazole		AD	Rat cortical neurons incubated with $A\beta_{25-35}$ peptide	Decreases neuronal Ca^{2+} dyshomeostasis (24 h incubation at 100 μM and 12 h incubation at 100μM, BD1047 at 10 μM blocked effect of afobazole). Reduces NO production (24 h incubation at 100 μM). Reduces neuronal death (72 h incubation at 100 μM). Inhibits the upregulation of the proapoptotic gene product Bax and caspase-3 (24 h incubation at 100 μM), preventing long-term downregulation of the antiapoptotic protein, Bcl-2 (48 h incubation at 100 μM).	[45]
			Rat primary cultures of microglia incubated with $A\beta_{25-35}$ peptide	Decreases microglial activation, reduces membrane ruffling (10 min incubation at 30 μM) and cell migration (migration was permitted during 2 h, afobazole was effective at 10 μM. BD-1047 at 10 μM blocked the effect of afobazole at 30 μM). Decreases microglial cell death (72 h incubation at 30 μM) by downregulation of expression of the proapoptotic protein Bax	[46]

Sigma1R agonist, NQO2 and MAO-A inhibitor			and caspase-3 (24 h incubation at 30 μM). Moderately increases Bcl-2 (24 h incubation at 30 μM). Normalizes intracellular calcium level (24 h incubation at 30 μM).	
	PD	6-OHDA mouse model	Normalizes motor function. Prevents decrease of DA in the striatum. Prevents the loss of TH + neurons in the substantia nigra. Doses and route of administration: 2.5 mg/kg daily i.p. for 14 days starting 30 minutes after the surgery. Pre-administration of BD-1047 at 3.0 mg/kg 30 min prior agonists abolished the action of afobazole.	[13]
			Prevents a decrease in the DA level. Doses and route of administration: 2.5 mg/kg for 14 days starting 30 min after the 6-OHDA injection.	[47]
			Facilitates motor behavior. Restores the intrastriatal DA content. Doses and route of administration: 2.5 mg/kg daily i.p. for 14 days starting 30 minutes after the surgery. Pre-administration of BD-1047 at 3.0 mg/kg 30 min prior agonists abolished the action of afobazole.	[14]

References

1. Meunier, J.; Ieni, J.; Maurice, T. The anti-amnesic and neuroprotective effects of donepezil against amyloid beta25-35 peptide-induced toxicity in mice involve an interaction with the sigma1 receptor. *British journal of pharmacology* **2006**, *149*, 998-1012, doi:10.1038/sj.bjp.0706927.
2. Maurice, T. Protection by sigma-1 receptor agonists is synergic with donepezil, but not with memantine, in a mouse model of amyloid-induced memory impairments. *Behavioural brain research* **2016**, *296*, 270-278, doi:10.1016/j.bbr.2015.09.020.
3. Goguadze, N.; Zhuravliova, E.; Morin, D.; Mikeladze, D.; Maurice, T. Sigma-1 Receptor Agonists Induce Oxidative Stress in Mitochondria and Enhance Complex I Activity in Physiological Condition but Protect Against Pathological Oxidative Stress. *Neurotoxicity research* **2019**, *35*, 1-18, doi:10.1007/s12640-017-9838-2.
4. Noh, M.Y.; Koh, S.H.; Kim, S.M.; Maurice, T.; Ku, S.K.; Kim, S.H. Neuroprotective effects of donepezil against Abeta42-induced neuronal toxicity are mediated through not only enhancing PP2A activity but also regulating GSK-3beta and nAChRs activity. *Journal of neurochemistry* **2013**, *127*, 562-574, doi:10.1111/jnc.12319.
5. Lahmy, V.; Long, R.; Morin, D.; Villard, V.; Maurice, T. Mitochondrial protection by the mixed muscarinic/sigma1 ligand ANAVEX2-73, a tetrahydrofuran derivative, in Abeta25-35 peptide-injected mice, a nontransgenic Alzheimer's disease model. *Frontiers in cellular neuroscience* **2014**, *8*, 463, doi:10.3389/fncel.2014.00463.
6. Lahmy, V.; Meunier, J.; Malmstrom, S.; Naert, G.; Givalois, L.; Kim, S.H.; Villard, V.; Vamvakides, A.; Maurice, T. Blockade of Tau hyperphosphorylation and Abeta(1)(-)(4)(2) generation by the aminotetrahydrofuran derivative ANAVEX2-73, a mixed muscarinic and sigma(1) receptor agonist, in a nontransgenic mouse model of Alzheimer's disease. *Neuropsychopharmacology* **2013**, *38*, 1706-1723, doi:10.1038/npp.2013.70.
7. Yang, R.; Chen, L.; Wang, H.; Xu, B.; Tomimoto, H.; Chen, L. Anti-amnesic effect of neurosteroid PREGS in Abeta25-35-injected mice through sigma1 receptor- and alpha7nAChR-mediated neuroprotection. *Neuropharmacology* **2012**, *63*, 1042-1050, doi:10.1016/j.neuropharm.2012.07.035.
8. Maurice, T.; Su, T.P.; Privat, A. Sigma1 (sigma 1) receptor agonists and neurosteroids attenuate B25-35-amyloid peptide-induced amnesia in mice through a common mechanism. *Neuroscience* **1998**, *83*, 413-428, doi:10.1016/s0306-4522(97)00405-3.
9. Borbely, E.; Varga, V.; Szogi, T.; Schuster, I.; Bozso, Z.; Penke, B.; Fulop, L. Impact of Two Neuronal Sigma-1 Receptor Modulators, PRE084 and DMT, on Neurogenesis and Neuroinflammation in an Abeta1-42-Injected, Wild-Type Mouse Model of AD. *International journal of molecular sciences* **2022**, *23*, doi:10.3390/ijms23052514.
10. Marrazzo, A.; Caraci, F.; Salinaro, E.T.; Su, T.P.; Copani, A.; Ronsisvalle, G. Neuroprotective effects of sigma-1 receptor agonists against beta-amyloid-induced toxicity. *Neuroreport* **2005**, *16*, 1223-1226, doi:10.1097/00001756-200508010-00018.

11. Wang, M.; Wan, C.; He, T.; Han, C.; Zhu, K.; Waddington, J.L.; Zhen, X. Sigma-1 receptor regulates mitophagy in dopaminergic neurons and contributes to dopaminergic protection. *Neuropharmacology* **2020**, 10.1016/j.neuropharm.2020.108360, 108360, doi:10.1016/j.neuropharm.2020.108360.
12. Francardo, V.; Bez, F.; Wieloch, T.; Nissbrandt, H.; Ruscher, K.; Cenci, M.A. Pharmacological stimulation of sigma-1 receptors has neurorestorative effects in experimental parkinsonism. *Brain* **2014**, *137*, 1998-2014, doi:10.1093/brain/awu107.
13. Voronin, M.V.; Kadnikov, I.A.; Voronkov, D.N.; Seredenin, S.B. Chaperone Sigma1R mediates the neuroprotective action of afobazole in the 6-OHDA model of Parkinson's disease. *Scientific reports* **2019**, *9*, 17020-17020, doi:10.1038/s41598-019-53413-w.
14. Kadnikov, I.A.; Verbovaya, E.R.; Voronkov, D.N.; Voronin, M.V.; Seredenin, S.B. Deferred Administration of Afobazole Induces Sigma1R-Dependent Restoration of Striatal Dopamine Content in a Mouse Model of Parkinson's Disease. *International journal of molecular sciences* **2020**, *21*, doi:10.3390/ijms21207620.
15. Watanabe, S.; Ilieva, H.; Tamada, H.; Nomura, H.; Komine, O.; Endo, F.; Jin, S.; Mancias, P.; Kiyama, H.; Yamanaka, K. Mitochondria-associated membrane collapse is a common pathomechanism in SIGMAR1- and SOD1-linked ALS. *EMBO Mol Med* **2016**, *8*, 1421-1437, doi:10.15252/emmm.201606403.
16. Gaja-Capdevila, N.; Hernandez, N.; Navarro, X.; Herrando-Grabulosa, M. Sigma-1 Receptor is a Pharmacological Target to Promote Neuroprotection in the SOD1(G93A) ALS Mice. *Frontiers in pharmacology* **2021**, *12*, 780588, doi:10.3389/fphar.2021.780588.
17. Mancuso, R.; Olivan, S.; Rando, A.; Casas, C.; Osta, R.; Navarro, X. Sigma-1R agonist improves motor function and motoneuron survival in ALS mice. *Neurotherapeutics* **2012**, *9*, 814-826, doi:10.1007/s13311-012-0140-y.
18. Prause, J.; Goswami, A.; Katona, I.; Roos, A.; Schnizler, M.; Bushuven, E.; Dreier, A.; Buchkremer, S.; Johann, S.; Beyer, C., et al. Altered localization, abnormal modification and loss of function of Sigma receptor-1 in amyotrophic lateral sclerosis. *Hum Mol Genet* **2013**, *22*, 1581-1600, doi:10.1093/hmg/ddt008.
19. Hyrskyluoto, A.; Pulli, I.; Tornqvist, K.; Ho, T.H.; Korhonen, L.; Lindholm, D. Sigma-1 receptor agonist PRE084 is protective against mutant huntingtin-induced cell degeneration: involvement of calpastatin and the NF-kappaB pathway. *Cell death & disease* **2013**, *4*, e646, doi:10.1038/cddis.2013.170.
20. Hall, H.; Iulita, M.F.; Gubert, P.; Flores Aguilar, L.; Ducatenzeiler, A.; Fisher, A.; Cuello, A.C. AF710B, an M1/sigma-1 receptor agonist with long-lasting disease-modifying properties in a transgenic rat model of Alzheimer's disease. *Alzheimers Dement* **2018**, *14*, 811-823, doi:10.1016/j.jalz.2017.11.009.
21. Fisher, A.; Bezprozvanny, I.; Wu, L.; Ryskamp, D.A.; Bar-Ner, N.; Natan, N.; Brandeis, R.; Elkon, H.; Nahum, V.; Gershonov, E., et al. AF710B, a Novel M1/sigma1 Agonist with Therapeutic Efficacy in Animal Models of Alzheimer's Disease. *Neurodegener Dis* **2016**, *16*, 95-110, doi:10.1159/000440864.
22. Ryskamp, D.; Wu, L.; Wu, J.; Kim, D.; Rammes, G.; Geva, M.; Hayden, M.; Bezprozvanny, I. Pridopidine stabilizes mushroom spines in mouse models of Alzheimer's disease by acting on the sigma-1 receptor. *Neurobiol Dis* **2019**, *124*, 489-504, doi:10.1016/j.nbd.2018.12.022.
23. Francardo, V.; Geva, M.; Bez, F.; Denis, Q.; Steiner, L.; Hayden, M.R.; Cenci, M.A. Pridopidine Induces Functional Neurorestoration Via the Sigma-1 Receptor in a Mouse Model of Parkinson's Disease. *Neurotherapeutics* **2019**, *16*, 465-479, doi:10.1007/s13311-018-00699-9.
24. Ponten, H.; Kullingsjo, J.; Sonesson, C.; Waters, S.; Waters, N.; Tedroff, J. The dopaminergic stabilizer pridopidine decreases expression of L-DOPA-induced locomotor sensitisation in the rat unilateral 6-OHDA model. *European journal of pharmacology* **2013**, *698*, 278-285, doi:10.1016/j.ejphar.2012.10.039.
25. Estevez-Silva, H.M.; Mediavilla, T.; Giacobbo, B.L.; Liu, X.; Sultan, F.R.; Marcellino, D.J. Pridopidine modifies disease phenotype in a SOD1 mouse model of amyotrophic lateral sclerosis. *The European journal of neuroscience* **2022**, *55*, 1356-1372, doi:10.1111/ejn.15608.
26. Squitieri, F.; Di Pardo, A.; Favellato, M.; Amico, E.; Maglione, V.; Frati, L. Pridopidine, a dopamine stabilizer, improves motor performance and shows neuroprotective effects in Huntington disease R6/2 mouse model. *J Cell Mol Med* **2015**, *19*, 2540-2548, doi:10.1111/jcmm.12604.
27. Shenkman, M.; Geva, M.; Gershoni-Emek, N.; Hayden, M.R.; Lederkremer, G.Z. Pridopidine reduces mutant huntingtin-induced endoplasmic reticulum stress by modulation of the Sigma-1 receptor. *Journal of neurochemistry* **2021**, *158*, 467-481, doi:10.1111/jnc.15366.
28. Naia, L.; Ly, P.; Mota, S.I.; Lopes, C.; Maranga, C.; Coelho, P.; Gershoni-Emek, N.; Ankarcrona, M.; Geva, M.; Hayden, M.R., et al. The Sigma-1 Receptor Mediates Pridopidine Rescue of Mitochondrial Function in Huntington Disease Models. *Neurotherapeutics* **2021**, *18*, 1017-1038, doi:10.1007/s13311-021-01022-9.
29. Nguyen, P.T.; Kimura, T.; Ho, S.A.; Tran, A.H.; Ono, T.; Nishijo, H. Ameliorative effects of a neuroprotective agent, T-817MA, on place learning deficits induced by continuous infusion of amyloid-beta peptide (1-40) in rats. *Hippocampus* **2007**, *17*, 443-455, doi:10.1002/hipo.20281.

30. Kimura, T.; Hong Nguyen, P.T.; Ho, S.A.; Tran, A.H.; Ono, T.; Nishijo, H. T-817MA, a neurotrophic agent, ameliorates the deficits in adult neurogenesis and spatial memory in rats infused i.c.v. with amyloid-beta peptide. *British journal of pharmacology* **2009**, *157*, 451-463, doi:10.1111/j.1476-5381.2009.00141.x.
31. Hirata, K.; Yamaguchi, H.; Takamura, Y.; Takagi, A.; Fukushima, T.; Iwakami, N.; Saitoh, A.; Nakagawa, M.; Yamada, T. A novel neurotrophic agent, T-817MA [1-{3-[2-(1-benzothiophen-5-yl) ethoxy] propyl}-3-azetidinol maleate], attenuates amyloid-beta-induced neurotoxicity and promotes neurite outgrowth in rat cultured central nervous system neurons. *The Journal of pharmacology and experimental therapeutics* **2005**, *314*, 252-259, doi:10.1124/jpet.105.083543.
32. Fukushima, T.; Nakamura, A.; Iwakami, N.; Nakada, Y.; Hattori, H.; Hoki, S.; Yamaguchi, H.; Nakagawa, M.; Terashima, N.; Narita, H. T-817MA, a neuroprotective agent, attenuates the motor and cognitive impairments associated with neuronal degeneration in P301L tau transgenic mice. *Biochemical and biophysical research communications* **2011**, *407*, 730-734, doi:10.1016/j.bbrc.2011.03.091.
33. Kim, W.S.; Fu, Y.; Dobson-Stone, C.; Hsiao, J.T.; Shang, K.; Hallupp, M.; Schofield, P.R.; Garner, B.; Karl, T.; Kwok, J.B.J. Effect of Fluvoxamine on Amyloid-beta Peptide Generation and Memory. *J Alzheimers Dis* **2018**, *62*, 1777-1787, doi:10.3233/JAD-171001.
34. Dalle, E.; Daniels, W.M.U.; Mabandla, M.V. Fluvoxamine maleate effects on dopamine signaling in the prefrontal cortex of stressed Parkinsonian rats: Implications for learning and memory. *Brain research bulletin* **2017**, *132*, 75-81, doi:10.1016/j.brainresbull.2017.05.014.
35. Caruso, G.; Grasso, M.; Fidilio, A.; Torrisi, S.A.; Musso, N.; Geraci, F.; Tropea, M.R.; Privitera, A.; Tascedda, F.; Puzzo, D., et al. Antioxidant Activity of Fluoxetine and Vortioxetine in a Non-Transgenic Animal Model of Alzheimer's Disease. *Frontiers in pharmacology* **2021**, *12*, 809541, doi:10.3389/fphar.2021.809541.
36. Sharma, A.; Mohammad, A.; Saini, A.K.; Goyal, R. Neuroprotective Effects of Fluoxetine on Molecular Markers of Circadian Rhythm, Cognitive Deficits, Oxidative Damage, and Biomarkers of Alzheimer's Disease-Like Pathology Induced under Chronic Constant Light Regime in Wistar Rats. *ACS chemical neuroscience* **2021**, *12*, 2233-2246, doi:10.1021/acschemneuro.1c00238.
37. Chung, Y.C.; Kim, S.R.; Park, J.Y.; Chung, E.S.; Park, K.W.; Won, S.Y.; Bok, E.; Jin, M.; Park, E.S.; Yoon, S.H., et al. Fluoxetine prevents MPTP-induced loss of dopaminergic neurons by inhibiting microglial activation. *Neuropharmacology* **2011**, *60*, 963-974, doi:10.1016/j.neuropharm.2011.01.043.
38. Shadfar, S.; Kim, Y.G.; Katila, N.; Neupane, S.; Ojha, U.; Bhurtel, S.; Srivastav, S.; Jeong, G.S.; Park, P.H.; Hong, J.T., et al. Neuroprotective Effects of Antidepressants via Upregulation of Neurotrophic Factors in the MPTP Model of Parkinson's Disease. *Molecular neurobiology* **2018**, *55*, 554-566, doi:10.1007/s12035-016-0342-0.
39. Liu, W.; Zhang, R.; Feng, H.; Zhu, H. Fluoxetine tunes the abnormal hippocampal oscillations in association with cognitive impairments in 6-OHDA lesioned rats. *Behavioural brain research* **2021**, *409*, 113314, doi:10.1016/j.bbr.2021.113314.
40. Grote, H.E.; Bull, N.D.; Howard, M.L.; van Dellen, A.; Blakemore, C.; Bartlett, P.F.; Hannan, A.J. Cognitive disorders and neurogenesis deficits in Huntington's disease mice are rescued by fluoxetine. *The European journal of neuroscience* **2005**, *22*, 2081-2088, doi:10.1111/j.1460-9568.2005.04365.x.
41. Chavant, F.; Deguil, J.; Pain, S.; Ingrand, I.; Milin, S.; Fauconneau, B.; Perault-Pochat, M.C.; Lafay-Chebassier, C. Imipramine, in part through tumor necrosis factor alpha inhibition, prevents cognitive decline and beta-amyloid accumulation in a mouse model of Alzheimer's disease. *The Journal of pharmacology and experimental therapeutics* **2010**, *332*, 505-514, doi:10.1124/jpet.109.162164.
42. Kandil, E.A.; Abdelkader, N.F.; El-Sayeh, B.M.; Saleh, S. Imipramine and amitriptyline ameliorate the rotenone model of Parkinson's disease in rats. *Neuroscience* **2016**, *332*, 26-37, doi:10.1016/j.neuroscience.2016.06.040.
43. Kumar, P.; Kalonia, H.; Kumar, A. Novel protective mechanisms of antidepressants against 3-nitropropionic acid induced Huntington's-like symptoms: a comparative study. *Journal of psychopharmacology (Oxford, England)* **2011**, *25*, 1399-1411, doi:10.1177/0269881110364269.
44. Maurice, T.; Volle, J.N.; Strehaiano, M.; Crouzier, L.; Pereira, C.; Kaloyanov, N.; Virieux, D.; Pirat, J.L. Neuroprotection in non-transgenic and transgenic mouse models of Alzheimer's disease by positive modulation of sigma1 receptors. *Pharmacol Res* **2019**, *144*, 315-330, doi:10.1016/j.phrs.2019.04.026.
45. Behensky, A.A.; Yasny, I.E.; Shuster, A.M.; Seredenin, S.B.; Petrov, A.V.; Cuevas, J. Afobazole activation of sigma-1 receptors modulates neuronal responses to amyloid-beta25-35. *The Journal of pharmacology and experimental therapeutics* **2013**, *347*, 468-477, doi:10.1124/jpet.113.208330.
46. Behensky, A.A.; Yasny, I.E.; Shuster, A.M.; Seredenin, S.B.; Petrov, A.V.; Cuevas, J. Stimulation of sigma receptors with afobazole blocks activation of microglia and reduces toxicity caused by amyloid-beta25-35. *The Journal of pharmacology and experimental therapeutics* **2013**, *347*, 458-467, doi:10.1124/jpet.113.208348.

47. Voronin, M.V.; Kadnikov, I.A.; Seredenin, S.B. Afobazole Restores the Dopamine Level in a 6-Hydroxydopamine Model of Parkinson's Disease. *Neurochem J* **2019**, *13*, 49-56, doi:10.1134/S1819712419010185.
